# NSD1-916aa encoded by *CircNSD1* contributes to AKI-to-CKD transition through inducing ferroptosis in tubular epithelial cells

**DOI:** 10.1172/jci.insight.189130

**Published:** 2025-07-15

**Authors:** Li Gao, Junsheng Zhang, Chaoyi Chen, Sai Zhu, Xianglong Wei, Guiqin Tang, Sheng Wang, Yukai Wang, Xinran Liu, Ling Jiang, Yonggui Wu

**Affiliations:** 1 Inflammation and Immune-Mediated Diseases Laboratory of Anhui Province, Anhui Institute of Innovative Drugs, School of Pharmacy, and; 2 Department of Nephropathy, The First Affiliated Hospital of Anhui Medical University, Hefei, China.; 3 Center for Scientific Research of Anhui Medical University, Hefei, China.; 4 Clinical Pathology Center, The First Affiliated Hospital of Anhui Medical University, Hefei, China.; 5 Anhui Public Health Clinical Center, Hefei, China.

**Keywords:** Metabolism, Nephrology, Chronic kidney disease

## Abstract

Acute kidney injury (AKI) is characterized by a rapid decline in renal function. In severe or recurrent cases, AKI can progress to chronic kidney disease (CKD), marked by renal inflammation and fibrosis. Despite the severity of these outcomes, early-stage diagnostic tools and pharmacological interventions for AKI-to-CKD progression remain limited. In this study, we examined circular RNA (circRNA) expression profiles in mouse renal cortex tissues 14 days after ischemia/reperfusion (I/R) injury using circRNA-Seq. The renal biopsy samples of patients after AKI exhibited reduced *CircNSD1* expression, which was inversely associated with inflammation and fibrosis. Overexpression of *CircNSD1* attenuated ferroptosis in vivo and in vitro, while slowing AKI-to-CKD progression. Mechanistically, *CircNSD1* downregulated *ACSL4* and *SLC39A14* expression through histone H3 lysine 36 (H3K36) methylation, a critical pathway regulating ferroptosis after AKI or hypoxia/reoxygenation (H/R) injury. Furthermore, we identified that *CircNSD1* encoded a NSD1-916aa peptide, which may functionally contribute to its observed effect. Collectively, these findings demonstrated that Circ*NSD1* may serve as a diagnostic and therapeutic target for early detection of AKI-to-CKD transition.

## Abstract 2

The Role of CircNSD1-Derived NSD1-916aa Protein in Promoting Ferroptosis and Facilitating the Transition from Acute Kidney Injury to Chronic Kidney Disease in Renal Tubular Epithelial Cells.

## Graphical abstract

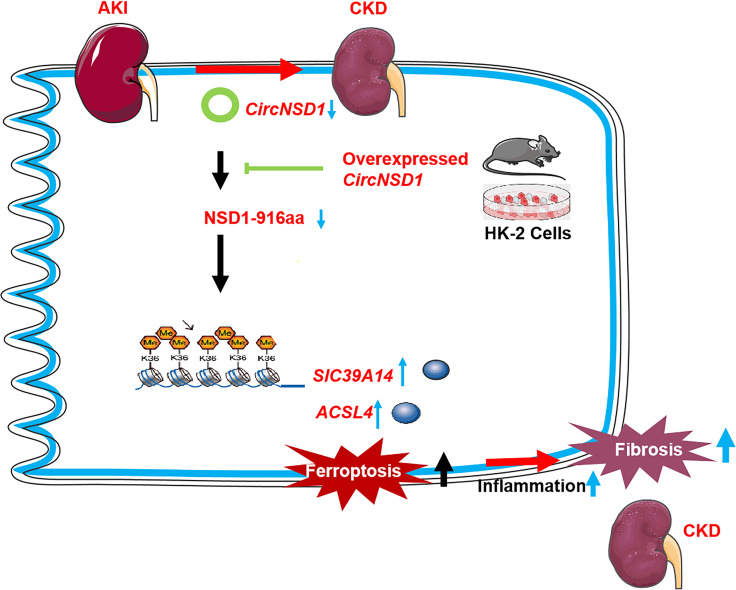

## Introduction

Acute kidney injury (AKI) is a critical clinical condition characterized by a sudden and pronounced deterioration in kidney function, often marked by proximal tubules damage and elevated serum creatinine and blood urea nitrogen (BUN) levels, and is associated with substantial morbidity and mortality rates (1). Furthermore, a considerable number of individuals with AKI experience progressive inflammation and fibrosis, ultimately developing chronic kidney disease (CKD) (2). Statistical analyses have indicated that patients with AKI are at a 2.67-fold higher risk of CKD and a 4.81-fold higher risk of end-stage renal disease (3, 4). Despite the severity of these outcomes, there is a lack of effective diagnostic tools and pharmacological interventions to treat AKI or mitigate its progression to CKD. Additionally, a comprehensive understanding of the molecular mechanisms underlying the AKI-to-CKD transition has yet to be fully elucidated, posing a challenge to the development of targeted clinical strategies (5).

Failed repair of renal epithelial tubular cells (PT cells) leads to a transition to a distinct proinflammatory state, which is implicated in the progression of fibrosis after AKI. The downregulation of genes involved in glutathione metabolism renders PT cells more susceptible to ferroptotic stress, intensifying the inflammatory response. The induction of high ferroptotic stress through genetic manipulation after injury aggravates inflammation and fibrosis, which are key drivers of AKI-to-CKD transition (6). Ferroptotic stress is characterized by an excessive accumulation of lipid reactive oxygen species, disruption in the metabolism of polyunsaturated fatty acids, and damage to mitochondrial function. This process participates in PT cell repair (6, 7). Emerging evidence highlights the protective effects of small-molecule inhibitors of ferroptosis, such as ferrostatin-1 (Fer-1) against AKI (8). However, the pathogenic mechanisms through which ferroptosis influences the transition from AKI to CKD remain poorly defined.

Advancements in next-generation sequencing have identified a multitude of uncharacterized transcripts. Among these, noncoding RNAs (ncRNAs) that lack an open reading frame (ORF) are traditionally considered nontranslatable. However, exonic circular RNAs (circRNAs) can harbor complete or partial ORFs corresponding to their linear parental genes, suggesting that certain circRNAs previously classified as noncoding may, in fact, be translatable. For instance, Circ-*ZNF609* encoded ZNF609-250aa to induce cell apoptosis and AKI by impairing the autophagy flux via AKT/mTOR-dependent mechanism (9). Similarly, circular *MTHFD2L* RNA-encoded CM-248aa inhibits gastric cancer progression by targeting the SET-PP2A interaction (10). In glioblastoma, a circular RNA (circMET, hsa_circ_0082002)-encoded MET variant has been shown to drive tumorigenesis (11). Despite growing insights into the roles of protein-coding circRNAs in various pathological contexts, their contributions to renal disease, particularly during the transition from AKI to CKD, remain poorly understood.

In this study, we identified a circRNA, designated *CircNSD1*, through high-throughput sequencing analysis of renal cortex tissues harvested from mice 14 days following ischemia/reperfusion (I/R) injury. *CircNSD1* expression was markedly downregulated in renal tissues from AKI-to-CKD mouse models, as well as in patient samples and hypoxia/reoxygenation–induced (H/R-induced) HK-2 cells. To elucidate the role of *CircNSD1* in the transition from AKI to CKD, we overexpressed *CircNSD1* both in vivo and in vitro. Subsequently, we conducted an untargeted liquid chromatography-tandem mass spectrometry (LC-MS/MS) lipidomics analysis to compare H/R-treated control cells with those overexpressing *CircNSD1*, aiming to uncover the molecular mechanisms. Furthermore, we explored the potential of *CircNSD1* to encode a bioactive peptide. Collectively, our findings suggest that *CircNSD1* may serve as a promising biomarker and therapeutic target for early intervention in the AKI-to-CKD transition.

## Results

### Injured epithelia drive inflammation and fibrosis contribution to AKI-to-CKD transition.

**Figure 1:**
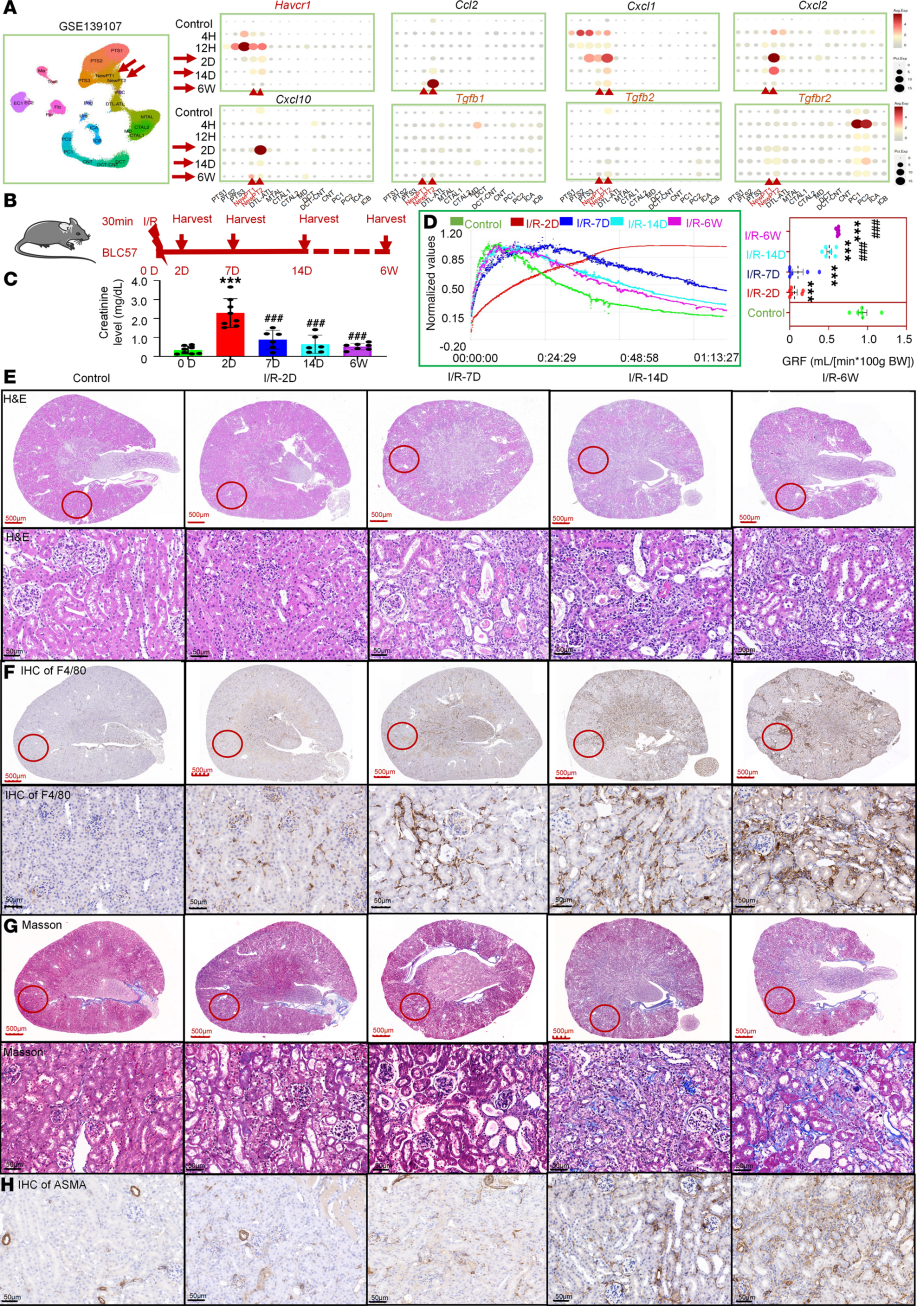
Fourteen-day I/R as a model for AKI-to-CKD transition in mice. (**A**) Reanalyzing a single-cell RNA-Seq dataset of mouse kidney (GSE139107) in KIT websites. (**B**) An illustration of AKI-to-CKD transition mouse model. (**C**) Serum creatinine levels. (**D**) GFR. (**E**) H&E staining. (**F**) IHC staining for F4/80. (**G**) Masson’s trichrome staining. (**H**) IHC staining for ASMA. *n* = 6–7. Scale bar: 100 μm (red), 50 μm (black). Data are shown as mean ± SEM, ***P < 0.001 compared with the control by 1-way ANOVA with Tukey’s post hoc test. ^###^*P* < 0.001 compared with the I/R-2D group by 1-way ANOVA with Tukey’s post hoc test.

To establish the mouse model of AKI-to-CKD transition, we analyzed a single-cell RNA-Seq dataset of mouse kidney published in PNAS in 2020 (GSE139107) (https://www.ncbi.nlm.nih.gov/geo/query/acc.cgi?acc=GSE139107). As shown in Figure 1A, cell clusters named NewPT1 and NewPT2 in PNAS (7), positive for the injury marker *Havcr1*, exhibited sustained high expression of inflammation-related genes (*Ccl2*, *Cxcl1*, *Cxcl2*, and *Cxcl10*) and fibrosis-related genes (*Tgfb1*, *Tgfb2* and *Tgfbr2*) from 2 days to 6 weeks after I/R. Figure 1B illustrates the AKI-to-CKD transition mouse model at various time points following I/R: 0 days, 2 days, 7 days, 14 days, and 6 weeks. As anticipated, serum creatinine, estimated glomerular filtration rate (GFR), H&E, and periodic acid–Schiff (PAS) staining revealed extensive tubular epithelial injury and rapid renal function decline at 2 days after I/R. Partial functional recovery was observed from 7 days to 6 weeks (Figure 1, C–E, and Supplemental Figure 1A; supplemental material available online with this article; https://doi.org/10.1172/jci.insight.189130DS1). Meanwhile, macrophage infiltration (F4/80), fibrotic deposition (Masson’s trichrome), and α–smooth muscle actin-marked (ASMA-marked) fibrotic areas were milder on day 7 but intensified by day 14 and persisted through week 6 (Figure 1, F–H, and Supplemental Figure 1B). These findings indicate that injured epithelial cells drive renal inflammation and fibrosis, which contributed to AKI-to-CKD transition. The subsequent inflammation and fibrosis mediated by injured epithelial cells can be observed substantially, suggesting that day 14 of ischemia is the optimal time point for AKI-to-CKD model.

### CircNsd1 expression is suppressed in mouse model of AKI-to-CKD transition, in H/R-induced epithelial cells and patients after AKI.

**Figure 2:**
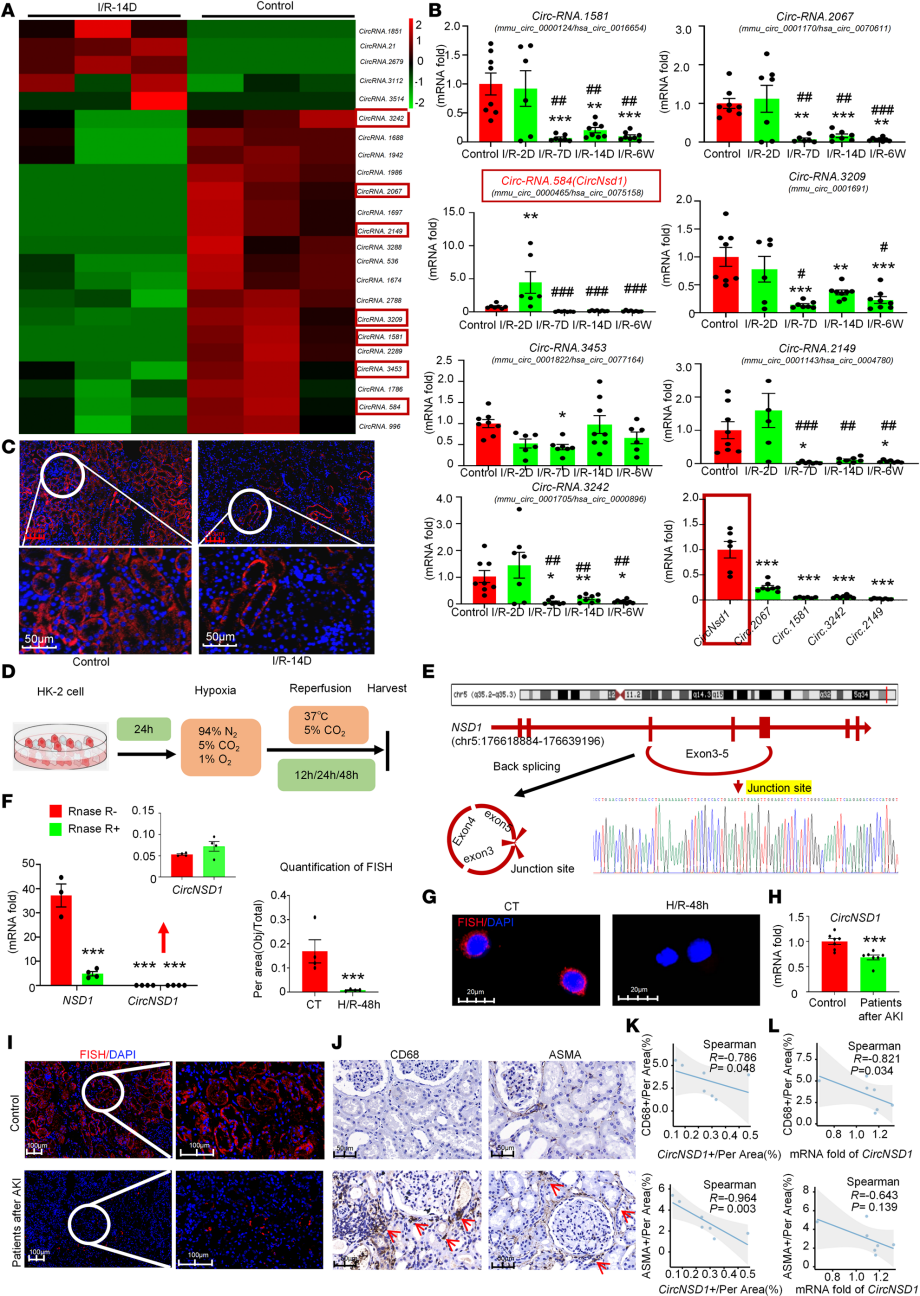
*CircNSD1* expression is suppressed in vivo, in vitro, and in patients. (**A**) Results of high-throughput circRNA-Seq in kidney tissues from control and I/R-14D mice. (**B**) Relative expression of circRNA detected by real time-PCR from 2 days to 6 weeks after I/R injury. Data are shown as mean ± SEM, *P < 0.05, **P < 0.01, ***P < 0.001 compared with the control or *CircNsd1* group by 1-way ANOVA with Tukey’s post hoc test. ^#^*P* < 0.05, ^##^*P* < 0.01, ^###^*P* < 0.001 compared with the I/R-2D group by 1-way ANOVA with Tukey’s post hoc test. (**C**) FISH assay for *CircNSD1*. Scale bar: 100 μm (red), 50 μm (white). (**D**) An illustration of H/R-induced HK-2 cells model. (**E**) Examination of *CircNSD1* in circBase (http://circbase.org/) via Sanger sequencing. (**F**) Real-time PCR analysis of *CircNSD1* and parental gene *NSD1*, digested by RNase R. Data are shown as mean ± SEM, ***P < 0.001 compared with RNase R^-^ RF1 group by 1-way ANOVA with Tukey’s post hoc test. (**G**) FISH assay for *CircNSD1* in HK-2 cells and H/R-induced HK-2 cells. Scale bar: 20 μm (white). (**H**) *CircNSD1* was detected in serum of control group and patients after AKI. Data are shown as mean ± SEM, ***P < 0.001 compared with Control group by Student’s *t* test. (**I**) FISH for *CircNSD1* in renal biopsies from controls and patients after AKI. Scale bar: 100 μm (white); 50 μm (black). (**J**) IHC for CD68^+^ reveals the inflammatory response, and ASMA reveals fibrosis in renal biopsies from controls and patients with AKI-to-CKD. Scale bar: 100 μm (black). (**K**) Correlation analysis between *CircNSD1* in renal biopsies and the inflammatory response (CD68^+^) or fibrosis (ASMA) in patients after AKI. (**L**) Correlation analysis between *CircNSD1* in serum and the inflammatory response (CD68^+^) or fibrosis (ASMA) in renal biopsies from patients after AKI.

To elucidate the role of circRNAs in the AKI-to-CKD transition, the renal cortex tissues from mice 14 days after I/R were subjected to high-throughput circRNA-Seq. Heatmap demonstrated the top 7 differentially expressed known circRNAs (Figure 2A). Subsequent validation via real-time PCR from 2 days to 6 weeks after I/R excluded circRNAs with insignificant expression changes or low homology during transition. *CircNsd1* emerged as the most dynamically altered circRNA with the highest basal expression (Figure 2B). FISH assay revealed that *CircNsd1* predominantly localized in the cytoplasm of tubular epithelial cells, with its expression decreasing in mice following I/R injury or cisplatin injury (Figure 2C and Supplemental Figure 1, C and D).

Cellular models utilizing HK-2 cells were established to further validate the expression of circRNAs that exhibited marked changes (Supplemental Figure 1E and Figure 2D). Notably, *CircNSD1* was confirmed as the circRNA with the most marked expression change and the highest level of basal expression level in vitro (Supplemental Figure 1F). *CircNSD1*, derived from the *NSD1* gene, is located on chromosome 5 (positions 176618884–176639196) and comprises exons 3–5. As depicted in Figure 2E, Sanger sequencing confirmed the presence of a head-to-tail splice junction characteristic of circRNAs, which matched the reference sequence for *CircNSD1* listed in circBase (http://circbase.org/). Considering that head-to-tail splicing products may be derived from genomic rearrangements or trans-splicing, the stability of *CircNSD1* was assessed by its resistance to digestion by RNase R (Figure 2F), a highly processive 3′–5′ exonuclease. The results indicated that the expression level of *CircNSD1* remained largely unaffected by RNase R treatment, whereas the expression of parental gene *NSD1* was substantially reduced. FISH corroborated *CircNSD1*’s cytoplasmic localization in HK-2 cells (Figure 2G). Furthermore, compared with normal controls, the expression of *CircNSD1* was decreased in serum and renal biopsies from patients after AKI (Figure 2, H and I). *CircNSD1* expression levels in renal tissues of patients after AKI were substantially reduced and showed a negative correlation with both renal inflammatory and fibrotic markers. Meanwhile, serum *circNSD1* levels were negatively correlated with inflammatory markers but showed no association with fibrosis — an observation that warrants further investigation with a large sample size (Figure 2, J–L).

### Overexpression of CircNSD1 resists AKI-to-CKD transition in vivo and in vitro.

**Figure 3:**
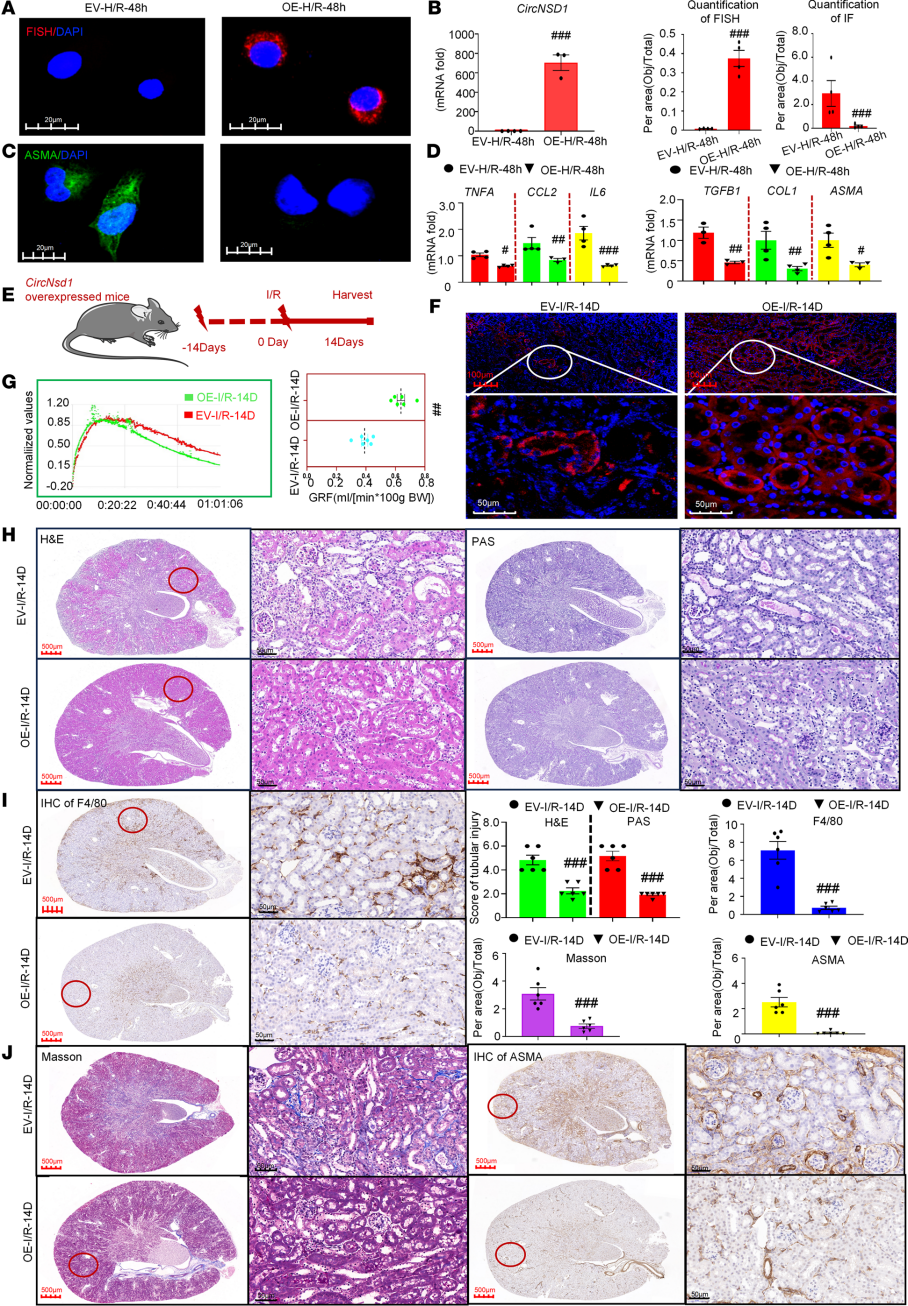
*CircNSD1* overexpression inhibits AKI-to-CKD transition. (**A**) FISH assay in *CircNSD1* overexpression cells. Scale bar: 20 μm (white). (**B**) Real-time PCR analysis of *CircNSD1* expression in *CircNSD1* overexpression cells. (**C**) Immunofluorescence for ASMA in *CircNSD1* overexpression cells. (**D**) Real-time PCR analysis of proinflammatory cytokines (*TNFA*, *CCL2*, and *IL6*) and fibrogenic genes (*TGFB1*, *COL1*, and *ASMA*) in H/R-48h induced control and *CircNSD1* overexpression cells. (**E**) Schematic illustrating the approach of overexpressing *CircNsd1* in mice. (**F**) FISH assay of *CircNsd1* expression in kidney tissues from control and *CircNsd1*-overexpressing I/R mice. Scale bar: 100 μm (red), 50 μm (white). (**G**) GFR in control and *CircNsd1*-overexpressing I/R mice. (**H**) Representative H&E staining and PAS staining pictures in kidney tissues from control and *CircNsd1*-overexpressing I/R mice. (**I**) IHC staining of F4/80^+^ macrophages in kidney tissues from control and *CircNsd1*-overexpressing I/R mice. (**J**) Representative Masson and IHC staining of ASMA pictures in kidney tissues from control and *CircNsd1*-overexpressing I/R mice. Scale bar: 500 μm (red), 50 μm (black). Data are shown as mean ± SEM, ^#^*P* < 0.05, ^##^*P* < 0.01, ^###^*P* < 0.001 compared with the EV-H/R-48h group or EV-I/R-14D group by Student’s t-test.

To investigate the role of *CircNSD1* in AKI-to-CKD transition, HK-2 cells were transfected with a lentiviral vector encoding *CircNSD1*. FISH assay revealed that *CircNSD1* was overexpressed in the cytoplasm of tubular epithelial cells (Figure 3A). Real-time PCR further confirmed *CircNSD1* overexpression in HK-2 (Figure 3B). Overexpression of *circNSD1* markedly reduced ASMA protein staining in HK-2 cells subjected to H/R injury (Figure 3C). Real-time PCR further confirmed decreased expression of inflammation-related genes (*TNFA*, *CCL2*, *IL6*) and fibrosis-related genes (*TGFB1*, *COL1*, *ASMA*) in *circNSD1*-overexpressed HK-2 cells, compared with control HK-2 cells after H/R injury (Figure 3D)

The role of *CircNsd1* was further validated in a murine model, and systemic *CircNsd1* overexpression was achieved in mice via tail vein injection of HBAAV2/9-*mmu_circ_0000465* (*CircNsd1*-OE; Figure 3E). Exogenous overexpression of *CircNsd1* in renal tissue was confirmed by real-time PCR and FISH (Figure 3F and Supplemental Figure 1G). PAS staining revealed no renal structural changes in sham-EV and sham-OE groups, confirming administration of HBAAV2/9-*circNsd1* via tail vein injection had no effect on normal renal structure (Supplemental Figure 1H). At 14 days after I/R injury, CircNsd1 overexpression group (OE-I/R-14D) markedly improved GFR compared with EV controls (EV-I/R-14D) (Figure 3G). H&E and PAS staining further corroborated reduced renal injury in the OE-I/R-14D group (Figure 3H). IHC showed diminished F4/80^+^ macrophage infiltration in renal epithelia of OE-I/R-14D mice (Figure 3I). Masson’s trichrome staining and ASMA IHC confirmed that *CircNsd1* overexpression substantially protected against I/R-induced AKI-to-CKD transition (Figure 3J). Collectively, these findings support that *CircNsd1* overexpression protected against I/R-induced renal dysfunction, inflammation, and fibrosis, thereby impeding the transition from AKI-to-CKD.

### Overexpression of CircNSD1 reduced ferroptosis in H/R-induced HK-2 cells and AKI-to-CKD mice.

**Figure 4:**
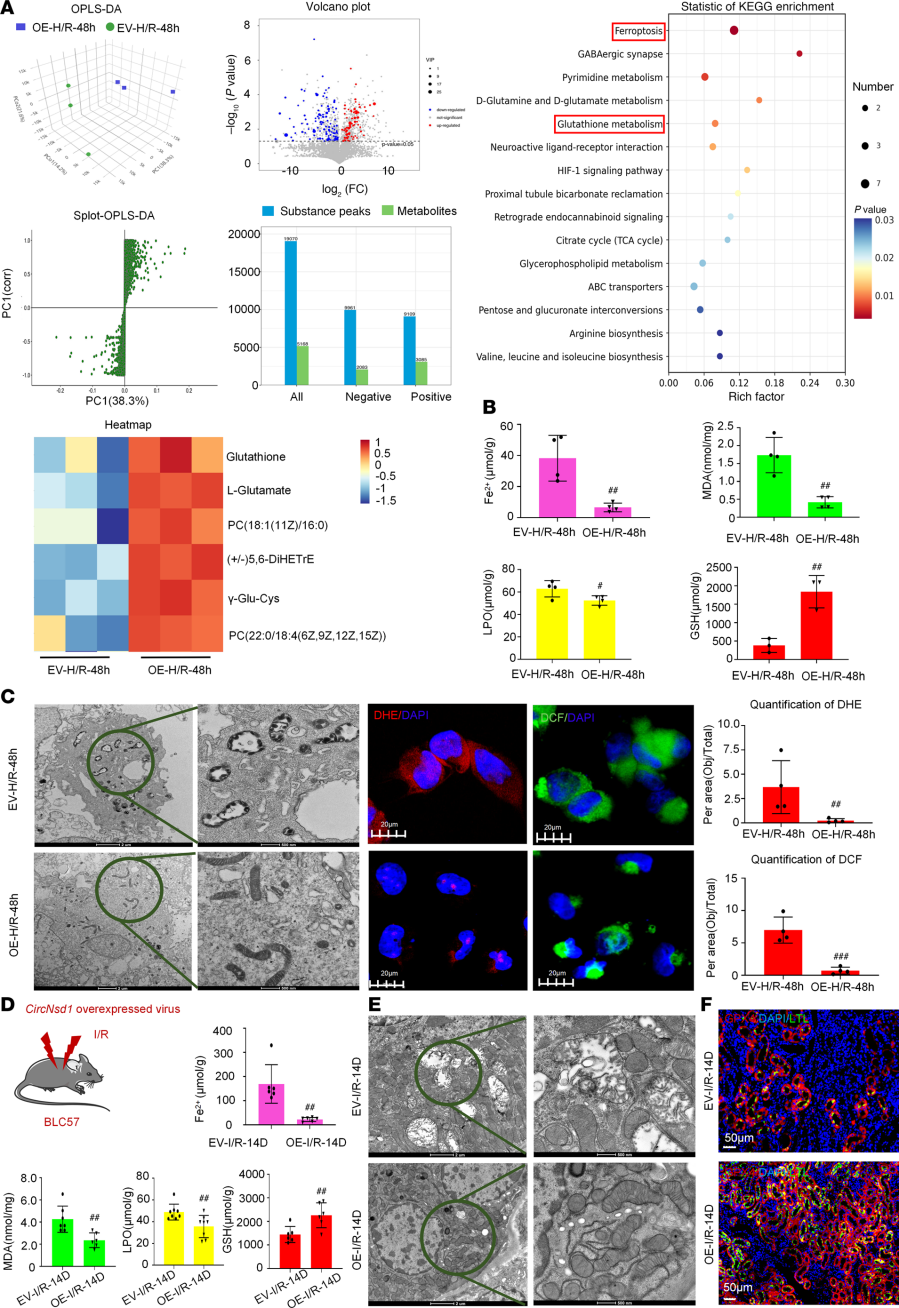
*CircNSD1* inhibits ferroptosis in AKI-to-CKD in vivo and in vitro. (**A**) Results LC-MS/MS lipidomics analysis of H/R-treated control and *CircNSD1* overexpression cells. (**B**) Levels of ferroptosis-related metabolites, including Fe^2+^, MDA, LPO, and GSH in vitro. (**C**) Electron microscopy, DCF assay and DHE assay in H/R-treated *CircNSD1* overexpression cells and controls. Scale bar: 2 μm (left); 500 nm (right); 20 μm (DHE and DCF staining). (**D**) An illustration of *CircNsd1* overexpression in mice model and metabolic (Fe^2+^, MDA, LPO and GSH) analysis in I/R-induced control and *CircNsd1* overexpression mice. (**E**) Representative electron micrographs of in kidney tissues from control and *CircNsd1*-overexpressing I/R mice. Scale bar: 2 μm (left); 500 nm (right). (**F**) Representative staining GPX4 in kidney tissues from control and *CircNsd1*-overexpressing I/R mice. Scale bar: 50 μm. Data are shown as mean ± SEM, ^#^*P* < 0.05, ^##^*P* < 0.01, ^###^*P* < 0.001, compared with EV-H/R-48h group or EV-I/R-14D by Student’s *t* test.

To decipher the underlying molecular mechanisms, untargeted LC-MS/MS lipidomics analysis of H/R-treated control and *CircNSD1* overexpression cells was performed. Orthogonal partial least squares discriminant analysis (OPLS-DA) identified 193 differentially abundant metabolites (variable importance in projection [VIP] ≥ 1, *P* < 0.05). Kyoto Encyclopedia of Genes and Genomes (KEGG) analysis revealed marked enrichment of ferroptosis-associated pathways, including ferroptosis and glutathione metabolism. Key ferroptosis-related metabolites included Glutathione, L-Glutamate, PC [18:1(11Z)/16:0], (±)5,6-DiHETrE, γ-Glu-Cys, and PC [22:0/18:4 (6Z,9Z,12Z,15Z)] (Figure 4A). Furthermore, *CircNSD1* overexpression in H/R-treated cells substantially restored levels of ferroptosis markers (Fe²^+^, malondialdehyde [MDA], lipid peroxidation [LPO], and glutathione [GSH]) compared with controls (Figure 4B). Electron microscopy, DCF assay, and DHE assay corroborated that overexpression of *CircNSD1* reduced ferroptosis under H/R condition (Figure 4C).

To validate these findings in vivo, we assessed ferroptosis-associated metabolites and proteins in I/R-induced control and *CircNsd1* overexpressing mice. Metabolic (Fe^2+^, MDA, LPO, and GSH) analysis, electron microscopy, and GPX4 IF assay showed that *CircNsd1* overexpression mitigated ferroptosis in AKI-to-CKD mice (Figure 4, D–F). These results support that overexpression of *CircNSD1* alleviated ferroptosis in H/R-induced HK-2 cells and AKI-to-CKD mice.

### Overexpression of CircNSD1 decreased Erastin-induced ferroptosis, inflammation, and fibrosis in HK-2 cells.

**Figure 5:**
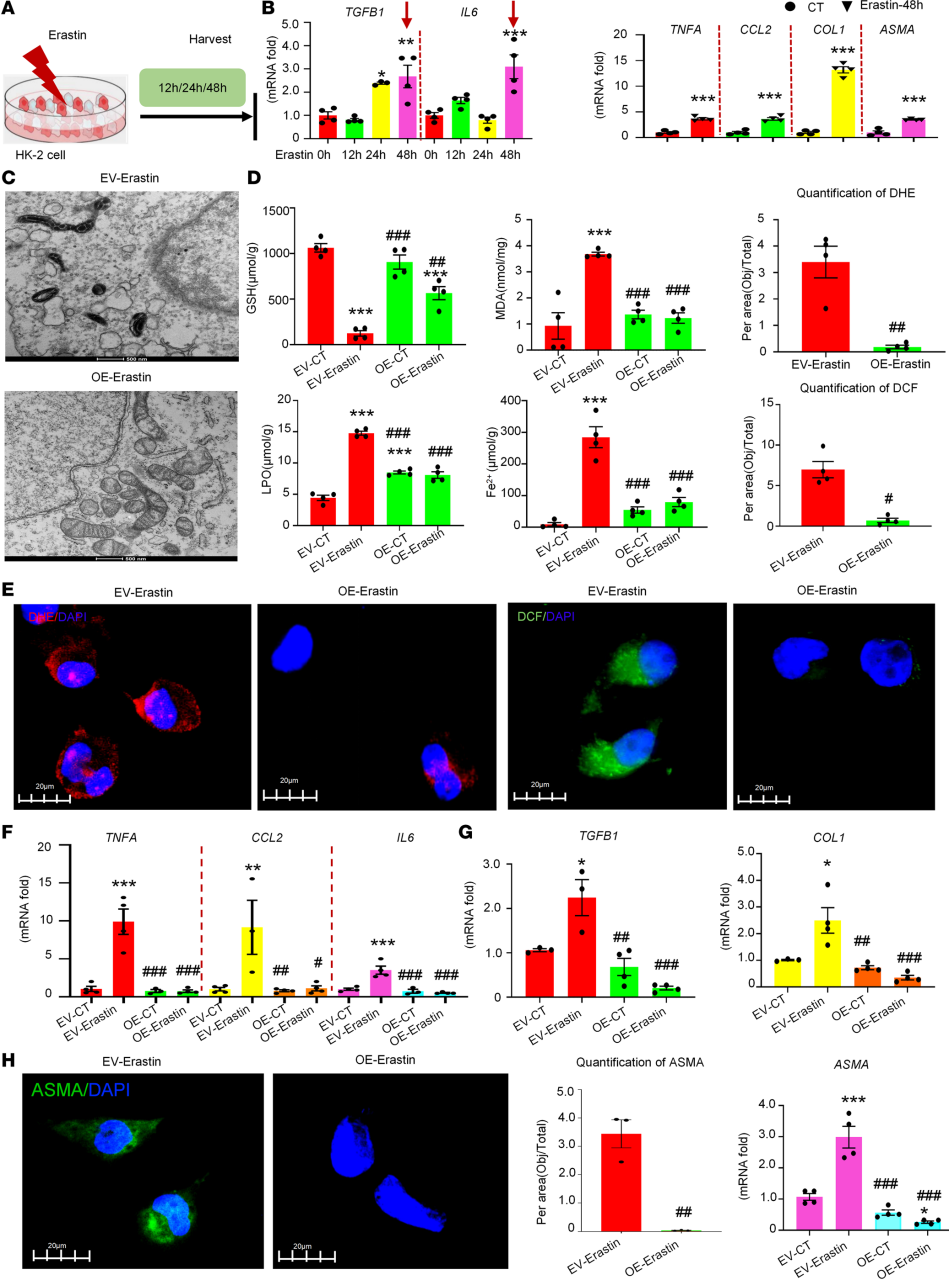
Overexpression of *CircNSD1* decreases ferroptosis, inflammation, and fibrosis in Erastin-induced HK-2 cells. (**A**) An illustration of Erastin-induced HK-2 cells model. (**B**) Real-time PCR analysis of inflammation-related genes (TNFA, CCL2, and IL6) and fibrosis-related genes (TGFB1, COL1, and ASMA) in HK-2 cells after 48 hours of Erastin induction. (**C**) Representative electron micrographs of control and CircNSD1-overexpressing cells induced by Erastin. Scale bar: 500nm. (**D**)Levels of ferroptosis-related metabolites,including Fe2+, MDA, LPO and GSH in cells. (**E**) DCF assay and DHE assay in Erastin-induced CircNSD1 overexpressing cells and controls. Scale bar: 20μm. (**F**) Real-time PCR analysis of proinflammatory cytokines (TNFA, CCL2 and IL6) in control and CircNSD1 overexpressing cells after 48 hours of Erastin induction. (**G**) Real-time PCR analysis of fibrosis-related genes (TGFB1,COL1 and ASMA) in Erastin-treated control and CircNSD1 overexpressing cells. (**H**) Representative immunofluorescence staining of ASMA pictures in control and CircNSD1 overexpressing cells treated with Erastin. Scale bar: 20 μm. Data are shown as mean ± SEM, *P < 0.05, **P < 0.01, ***P < 0.001 compared with the control by 1-way ANOVA with Tukey’s posthoc test. ^#^*P* < 0.05, ^##^*P* < 0.01, ^###^*P* < 0.001 compared with the EV-Erastain group by 1-way ANOVA with Tukey’s post hoc test.

To investigate whether the role of *CircNSD1* in inflammation and fibrosis via ferroptosis pathway, control and *CircNSD1*-overexpressing cells were treated with the ferroptosis inducer Erastin. Real-time PCR indicated that treatment of HK-2 cells with 5 μM Erastin for 48 hours led to increased expression of inflammation-related genes (*TNFA*, *CCL2*, and *IL6*) and fibrosis-related genes (*TGFB1*, *COL1*, and *ASMA*) (Figure 5, A and B). Subsequent assays, including electron microscopy; metabolic analysis of Fe^2+^, MDA, LPO, and GSH level; and DHE/DCF fluorescence assay, collectively suggested that overexpression of *CircNSD1* marked reduced Erastin-induced ferroptosis (Figure 5, C–E). Concurrently, real-time PCR showed a decrease in the expression of inflammation-related genes (*TNFA*, *CCL2*, and *IL6*) and fibrosis-related genes (*TGFB1*, *COL1*, and *ASMA*) in *CircNSD1*-overexpressing cells following Erastin treatment (Figure 5, F and G). Furthermore, IF staining of ASMA further confirmed a diminished fibrotic response in the overexpression group compared with the control group (Figure 5H). Collectively, these findings support that overexpression of *CircNSD1* alleviated inflammation and fibrosis induced by ferroptosis in vitro.

### Overexpression of CircNSD1 downregulated ACSL4 and SLC39A14 by H3K36 methylation.

**Figure 6:**
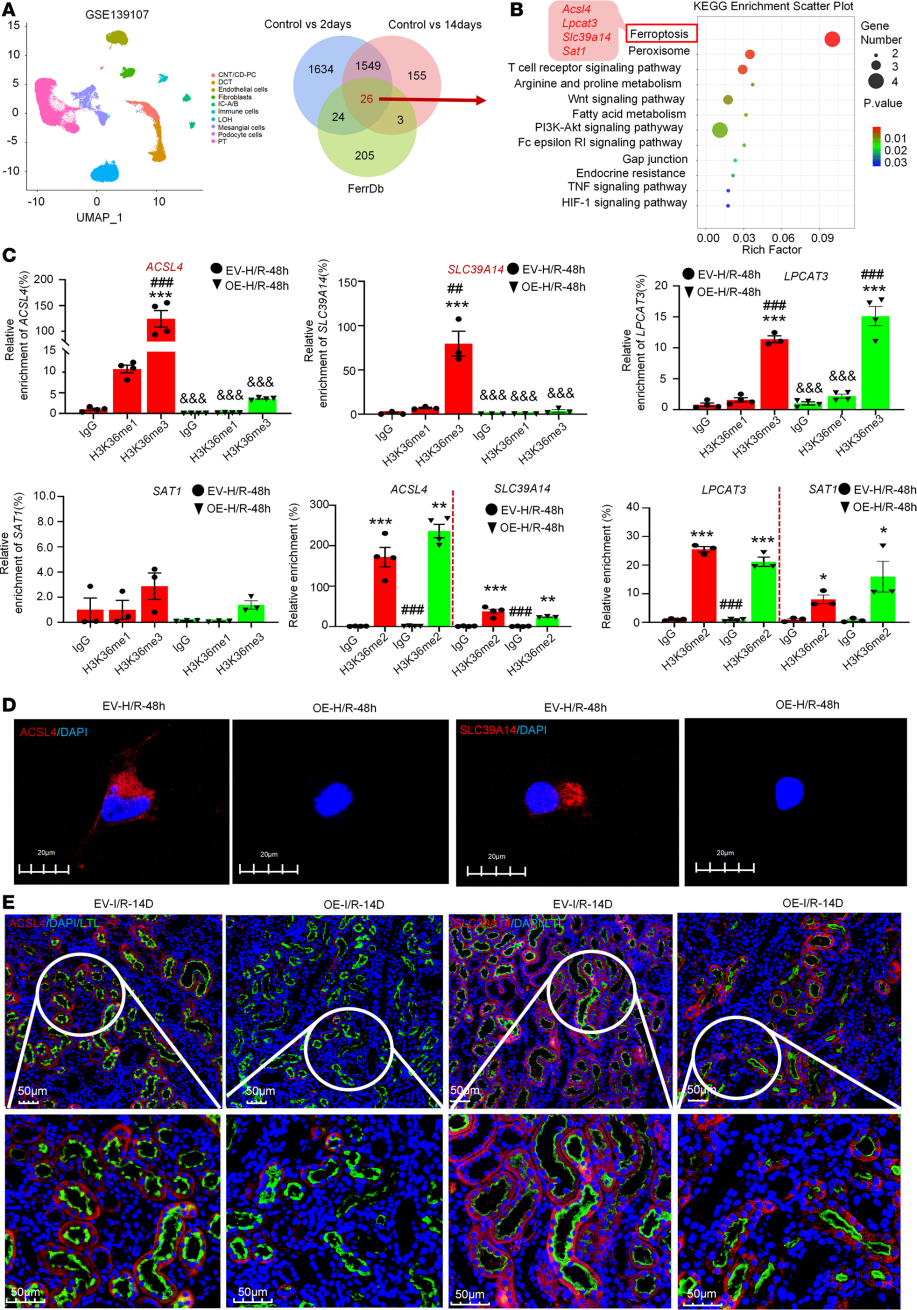
Overexpression of *CircNSD1* downregulated *ACSL4* and *SLC39A14* by H3K36 methylation. (**A**) Reanalyzing a single-cell RNA-Seq dataset of mouse kidney (GSE139107). Venn diagram showing the intersection analysis of genes changed in I/R-2D and I/R-14D with genes associated with ferroptosis. (**B**) KEGG pathway analysis of overlapping genes. (**C**) ChIP assay detecting the H3K36me modification of *ACSL4*, *LPCAT3*, *SLC39A14*, and *SAT1* regulated by *CircNSD1* in control and CircNSD1 overexpressed cells treated with H/R-48h. (**D**) Representative immunofluorescence staining of ACSL4 and SLC39A14 in control and *CircNSD1* overexpressed cells treated with H/R-48h. Scale bar: 20 μm. (**E**) Immunofluorescence staining of ACSL4 and LTL in kidney tissues from control and *CircNsd1*-overexpressing I/R mice. Immunofluorescence staining of SLC39A14 and LTL in kidney tissues from control and *CircNsd1*-overexpressing I/R mice. Scale bar: 50 μm. Data are shown as mean ± SEM, *P < 0.05, **P < 0.01, ***P < 0.001 compared with the IgG-EV-H/R-48h group by 1-way ANOVA with Tukey’s post hoc test. ^###^*P* < 0.001 compared with H3K36me1-EV-H/R-48h group or H3K36me2-EV-H/R-48h group by 1-way ANOVA with Tukey’s post hoc test. ^&&&^*P* < 0.001 compared with H3K36me3-EV-H/R-48h group by 1-way ANOVA with Tukey’s post hoc test.

Ferroptosis, a nonapoptotic form of cell death, plays a critical role in the AKI-to-CKD transition (12). During this transition, key ferroptosis-related proteins such as GPX4 and ACSL4 exhibit dynamic changes. Immunofluorescence, real-time PCR, and spatial transcriptomics datasets (GSE139107) reveal decreased GPX4 expression in early AKI stages, with partial restoration during CKD progression, alongside persistently elevated ACSL4 levels in renal epithelial cells (Supplemental Figure 1, I and J). To examine the direct mechanisms by which *CircNsd1* regulates ferroptosis, reanalysis of single-cell datasets (GSE139107) was performed to identify differentially expressed genes (log_2_FC > 0.26 and *P* < 0.05) at 2 and 14 days after AKI compared with the control group (Figure 6A). Intersection with FerrDb (a ferroptosis database; http://www.zhounan.org/ferrdb/current/) ferroptosis-related genes yielded 26 candidates. KEGG pathway analysis of the resulting 26 genes revealed that *Acsl4*, *Lpcat3*, *Slc39a14*, and *Sat1* are involved in the ferroptosis pathway (Figure 6B). Therefore, we focused on investigating the regulation of *Acsl4*, *Lpcat3*, *Slc39a14*, and *Sat1* by *CircNsd1*.

The parent gene of *CircNSD1*, *NSD1*, encodes a protein involved in histone H3 lysine 36 dimethylation (H3K36me) (13, 14). The ChIP assay further revealed a reduced enrichment of H3K36me3, in contrast to H3K36me1 and H3k36me2, within the promoter regions of *ACSL4* and *SLC39A14* in *CircNSD1*-overexpressing cells subjected to H/R-induced injured cells. These findings suggest that overexpression of *CircNSD1* may attenuate *ACSL4* and *SLC39A14* DNA H3K36 methylation by H3K36me3 (Figure 6C). IF confirmed *CircNSD1* overexpression substantially reduced ACSL4 and SLC39A14 expression in H/R-injured cells and AKI-to-CKD mice (Figure 6, D and E). Together, these findings demonstrate that *CircNSD1* overexpression alleviates ferroptosis by downregulating *ACSL4* and *SLC39A14* in vitro and in vivo.

### CircNSD1 encoded a 916aa peptide termed NSD1-916aa.

**Figure 7:**
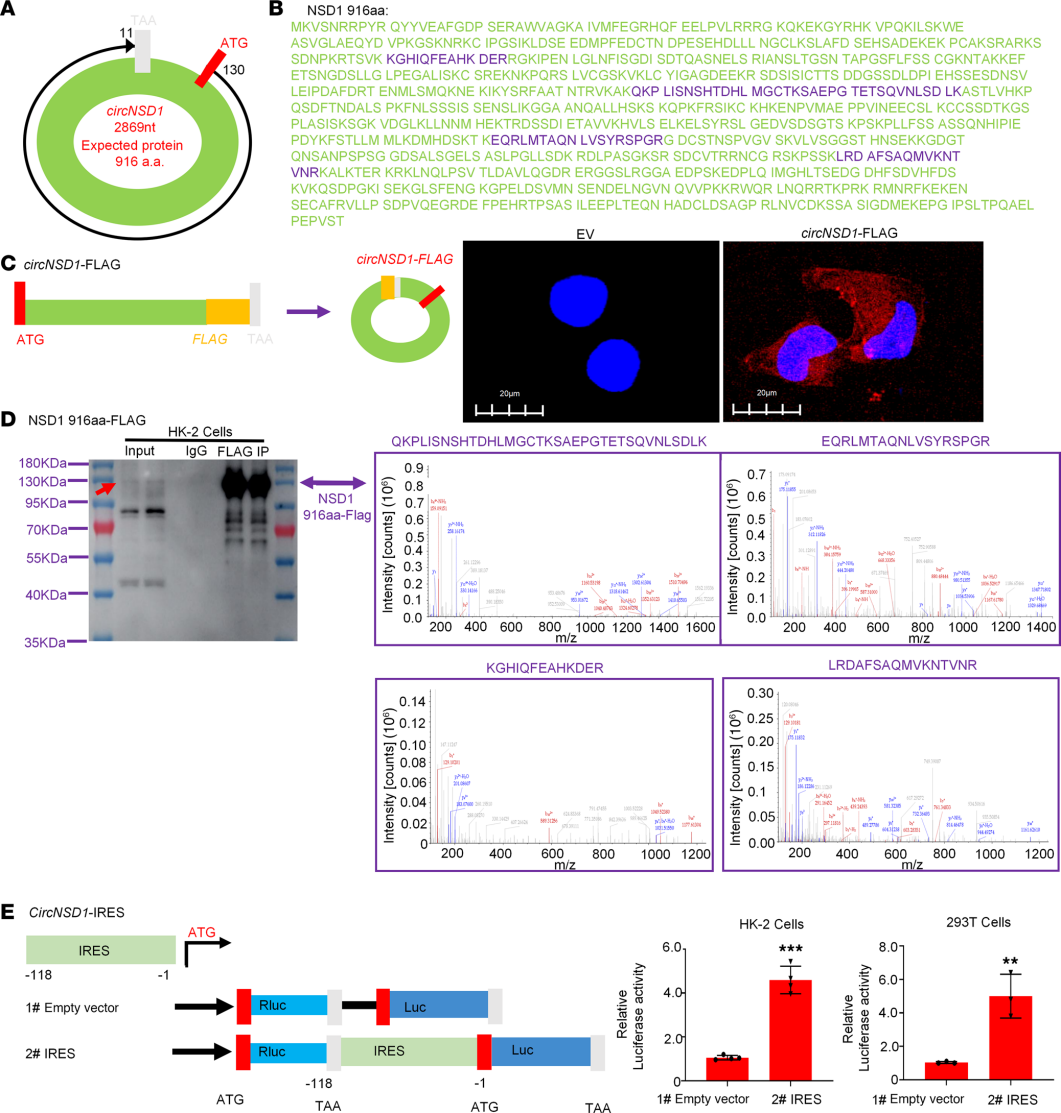
*CircNSD1* encodes a 916aa peptide. (**A**) The 2869 nt *circNSD1* is potentially translated into a 916aa protein (NSD1-916aa). (**B**) The sequences indicate the unique amino acids formed by the spanning junction ORF. (**C**) An illustration of overexpression of FLAG-tagged *circNSD1*. Immunofluorescence for NSD1-916aa in HK-2 cells. (**D**) Western blot analysis showing NSD1-916aa in HK-2 cells and the MS results of HK-2 cells revealing 4 unique sequences of NSD1-916aa. (**E**) Luciferase reporter assay indicating IRES activity of the *CircNSD1* UTR exists in HK-2 and 293T cells. Scale bar: 20 μm (white). Data are shown as mean ± SEM, **P < 0.01, ***P < 0.001 compared with the EV group by Student’s *t* test.

To further explore whether *CircNSD1* functioned by encoding a peptide — consistent with emerging evidence of protein-coding potential in certain circRNAs (9) — we performed immunoprecipitation of FLAG-tagged *CircNSD1* and luciferase-reporter assays in HK-2 and 293T cells. As predicted by circRNA Db (http://reprod.njmu.edu.cn/circrnadb/), a comprehensive repository for human circRNAs with protein-coding annotations (15), *CircNSD1* initiates translation at the ATG start codon and terminates at the TAA stop codon (Figure 7A). This circRNA contains an ORF spanning 2,869 nucleotides, encoding a protein of 916 amino acids protein (916aa). Thus, we named the predicted protein NSD1-916aa, and its sequence is shown in Figure 7B. To further characterize NSD1-916aa, we performed immunofluorescence and MS following the immunoprecipitation and overexpression of FLAG-tagged *circNSD1*. Immunofluorescence staining of Flag confirmed NSD1-916aa expression in the cytoplasm and nucleus of HK-2 cells (Figure 7C). The MS results of HK-2 cells successfully identified 4 unique sequences of NSD1-916aa, including “QKPLISNSHTDHLMGCTKSAEPGTETSQVNLSDLK,” “EQRLMTAQNLVSYRSPGR,” “KGHIQFEAHKDER,” and “LRDAFSAQMVKNTVNR.” FLAG-tagged *CircNSD1* was also transfected into 293T cells, with MS confirming the peptide sequence “QKPLISNSHTDHLMGCTKSAEPGTETSQVNLSDLK.” These results demonstrate that *CircNSD1* is translated into the NSD1-916aa protein (Figure 7D and Supplemental Figure 2A).

Meanwhile, to validate the internal ribosome entry site (IRES) activity of *CircNSD1*, a luciferase reporter assay was performed in HK-2 and 293T cells. The promoter drives transcription of firefly luciferase (Fluc) and Renilla luciferase coding regions, with the spacer in bicistronic constructs replaced by either an empty vector or the *CircNSD1* untranslated region (UTR) containing the IRES. Results show that intact IRES sequences could trigger Fluc activity, indicating that IRES activity of the *CircNSD1* UTR exists (Figure 7E). Collectively, these results confirm that *CircNSD1* encoded a 916aa peptide.

## Discussion

**Figure 8:**
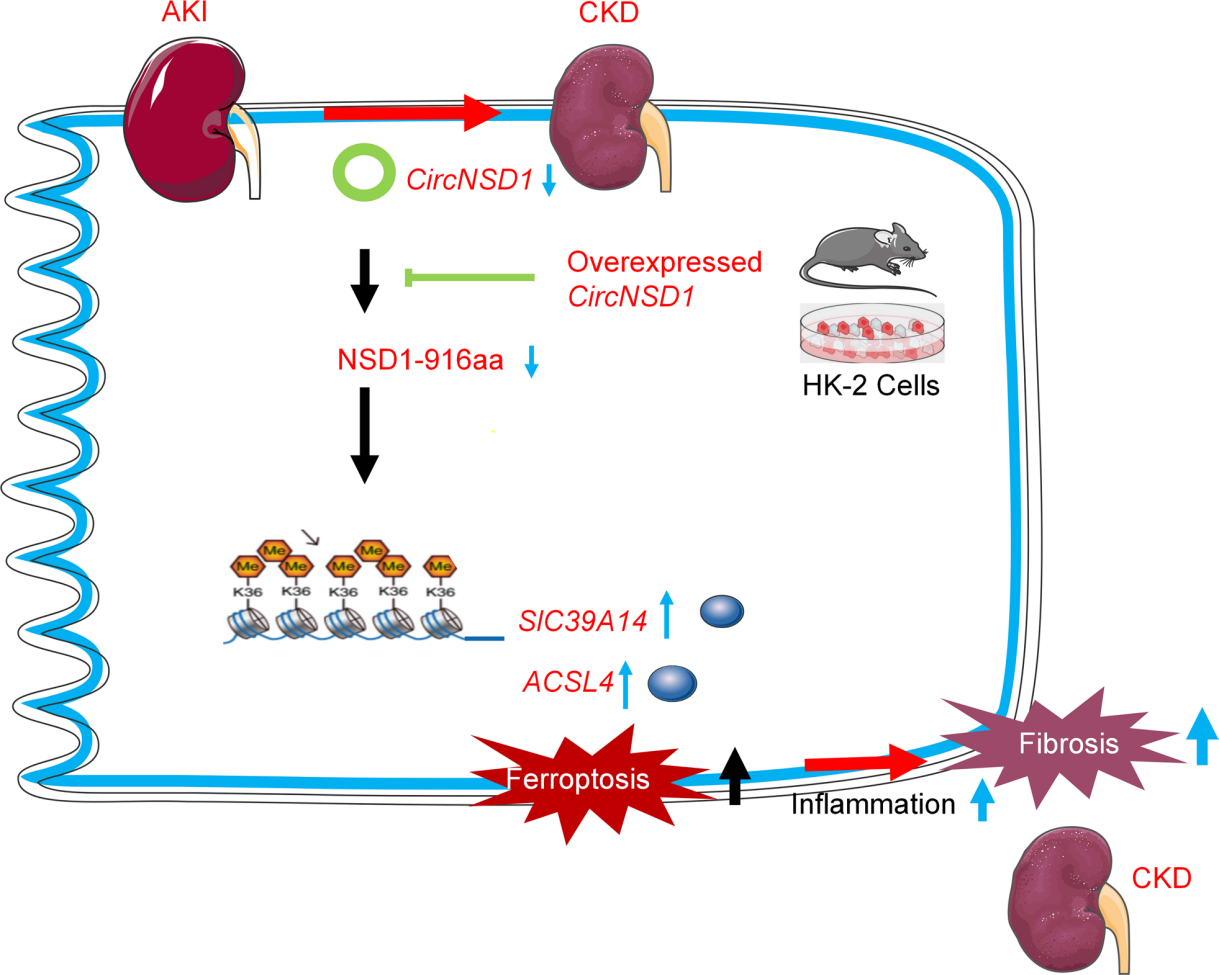
Graphical abstract. During the transition from AKI to CKD, *CircNSD1* expression decreases and is negatively correlated with inflammation and fibrosis. In vivo and in vitro, overexpression of *CircNSD1* mitigates ferroptosis in renal tubular epithelial cells and slows the progression of AKI to CKD. Mechanically, *CircNSD1* can encode a NSD1-916aa peptide, which downregulates *ACSL4* and *SLC39A14* through H3K36 methylation and regulates ferroptosis, inflammation, and fibrosis in AKI to CKD.

AKI is a clinical syndrome associated with high morbidity and mortality and often progresses to CKD, particularly in cases of medical negligence. Due to the unclear pathogenesis of AKI, targeted treatments remain limited, with hemodialysis being the primary intervention. In the present study, we identified a circRNA, named *CircNSD1*, which we propose as a potential diagnostic biomarker for AKI-to-CKD transition. *CircNSD1* was identified though high-throughput circRNA-Seq of renal cortex tissues, and its downregulation was observed in tissues of AKI-to-CKD mouse model, patients after AKI, and H/R-induced HK-2 cells. Our findings indicate that overexpression of *CircNSD1* reduced ferroptosis in vivo and in vitro, thereby slowing the progression of AKI-to-CKD transition. We provide evidence that overexpression of *CircNSD1* downregulates *ACSL4* and *SLC39A14* though the methylation of H3K36, a key mechanism by which *CircNSD1* mitigates ferroptosis induced by AKI or H/R. Furthermore, we found that *CircNSD1* might encoded a NSD1-916aa peptide, which could have functional implications in its effects. Collectively, these results demonstrate that *CircNSD1* is a promising diagnostic and therapeutic target for the early detection of AKI-to-CKD transition (Figure 8).

In this research, we investigated the role of epithelial-specific *CircNSD1* in the AKI-to-CKD transition. circRNAs are highly stable, widely expressed across various cell types, and conserved across species and tissues (16–18). Studies have demonstrated their critical involvement in the pathogenesis, progression, diagnosis, and prognosis of AKI (19, 20). Notably, circRNAs exhibit dynamic, spatiotemporally regulated expression patterns in kidney-related diseases. For instance, Xu et al. reported that circ-AKT3 exacerbates renal I/R injury by sponging miR-144-5p and activating the *Wnt/bcatenin* pathway and oxidative stress (21). Cheng et al. systematically analyzed the competing endogenous RNA mechanism involving circRNAs and long ncRNAs in a rat model of contrast-induced AKI (CI-AKI), offering insights into the pathogenesis of CI-AKI (22). Although alterations in circRNA expression have been observed in AKI, direct mechanistic links of circRNAs in the AKI-to-CKD transition remain unexplored. This study is the first to our knowledge to reveal that *CircNSD1* regulated epithelial injury, with its expression levels being inversely associated with inflammation and fibrosis in AKI-to-CKD patients. These findings suggest that *CircNSD1* may serve as a potential diagnostic biomarker for AKI-to-CKD transition.

Growing evidence implicates ferroptosis as a key driver of AKI (23–25). Ferroptosis has been confirmed in mouse models of AKI induced by nephrotoxic drugs and rhabdomyolysis (26–28). Moreover, ferroptotic stress has been identified as a key contributor to the pathogenesis of AKI and its subsequent transition to CKD. Research from Duke University School of Medicine (Durham, North Carolina, USA) has elucidated the molecular mechanisms underlying this transition. Following AKI-induced injury, proximal tubular cells adopt a proinflammatory state that promotes fibrosis. This inflammatory phenotype is transient in cases of mild injury, allowing for a return to the basal state without fibrosis development. However, in instances of severe injury, the inflammatory state becomes chronic, leading to ongoing inflammation and fibrosis. The downregulation of genes involved in glutathione metabolism makes PT cells more susceptible to ferroptotic stress, intensifying the inflammatory response. Genetic induction of high ferroptotic stress after injury exacerbates inflammation and fibrosis, pivotal drivers of AKI-to-CKD transition (6). Our experiments corroborated these findings, demonstrating dynamic changes in ferroptosis markers (e.g., GPX4 and ACSL4) during AKI-to-CKD progression (Supplemental Figure 1, I and J). Collectively, these data underscore ferroptosis as a pathogenic mechanism in this transition. Additionally, untargeted LC-MS/MS analysis of H/R-treated control cells and those overexpressing *CircNSD1* revealed a notable enrichment of differential metabolites in the ferroptosis and glutathione metabolism pathways. Furthermore, ChIP assay demonstrated reduced enrichment of H3K36me3 and H3K36me1 at the promoter regions of *ACSL4* and *SLC39A14* in *CircNSD1*-overexpressing cells subjected to H/R-induced injury. These results indicate that the overexpression of *CircNSD*1 may mitigate ferroptosis through the modulation of *ACSL4* and *SLC39A14* DNA H3K36 methylation patterns during the AKI-to-CKD transition.

Although circRNAs have traditionally been classified as ncRNAs, recent studies indicate that they still have translational functions, and a few circRNAs can be translated into functional proteins under the regulation of methylation modification and participate in a variety of cellular processes (29–31). Notably, protein-coding circRNAs have garnered attention for their involvement in pathogenesis, including muscle atrophy, gastric cancer, AKI, and so on (10, 32–34). For instance, Circ-ZNF609 encoded ZNF609-250aa to induce cell apoptosis and AKI by impairing the autophagy flux via AKT/mTOR-dependent mechanism (9). Despite growing insights into the roles of protein-coding circRNAs in various diseases, their involvement in renal disease progression remain poorly characterized, particularly in the context of the AKI-to-CKD transition. In this study, we identified a protein-coding circRNA, *CircNSD1*, which initiates translation at the start codon ATG and terminates at the stop codon TAA, encompassing an ORF of 2869 nucleotides. This ORF encodes a protein of 916aa in length, referred to as NSD1-916aa. Our findings suggest that NSD1-916aa, the translational product of *CircNSD1*, may play a pivotal role in mitigating ferroptosis in the pathogenesis of AKI and CKD.

NSD1, a H3K36 methyltransferase, regulates gene expression through its catalytic activity on histone H3 lysine 36 (H3K36). NSD1 catalyzes the mono- and dimethylation of H3K36, serving as substrates for trimethylation by SETD2. Through interaction with promoter regions and RNA polymerase II (RNAPII), NSD1 modulates transcriptional activity and promotes gene expression by facilitating RNAPII-mediated elongation (35). In this study, overexpression of *CircNSD1* reduced H3K36 methylation at *ACSL4* and *SLC39A14* loci during AKI-to-CKD progression. Overexpression of *CircNSD1* exerts effects opposing those of their corresponding linear parent genes (NSD1), potentially due to NSD1-916aa’s interaction with Cullin-associated nedd8-dissociated protein (CAND1) in HK-2 and 293T cells (Supplemental Figure 2, A and B). This interaction between may attenuate the ubiquitination process catalyzed by Cullin 1, a core component of the SCF (SKP1, CUL1/CDC53, F box proteins) E3 ubiquitin ligase complex (36). Stabilization of NSD1-916aa may be essential for *CircNSD1*’s ability to suppress H3K36 methylation at *ACSL4* and *SLC39A14* promoters. This epigenetic modulation is critical for mitigating ferroptosis in the context of AKI and subsequently slow the progression to CKD.

In conclusion, our study identifies *CircNSD1* as a potential diagnostic biomarker for the AKI-to-CKD transition. *CircNSD1* overexpression attenuate ferroptosis in both in vivo and in vitro models, thereby slowing the progression of AKI to CKD. Additionally, the overexpression of *CircNSD1* was found to downregulate *ACSL4* and *SLC39A14* through H3K36 methylation, a critical epigenetic modification that represents a key mechanism in the role of *CircNSD1* in ferroptosis induced by AKI or H/R injury. Furthermore, our findings reveal that *CircNSD1* encodes a NSD1-916aa peptide, which likely contributes to its functional role in AKI pathophysiology. Collectively, these findings advance our understanding of *CircNSD1*’s molecular mechanisms in AKI and highlight its therapeutic potential for halting CKD progression.

## Methods

### Sex as a biological variable.

Male C57BL/6J mice (purchased from the Experimental Animal Center of Anhui Medical University) were utilized in this study. Our study exclusively examined male mice in this model of AKI to CKD. It is unknown whether the findings are relevant for female mice.

### Human samples.

Patients with nephropathies who underwent renal biopsies in the Nephrology Department between 2022 and 2024 were screened for inclusion (37, 38). Exclusion criteria for the post-AKI group included: (a) immune-mediated nephropathy; (b) patients diagnosed with AKI who progressed to renal failure or even death; and (c) incomplete clinical data or absence of key laboratory findings.

Inclusion criteria for the post-AKI group were as follows. (a) Diagnosis of AKI per Kidney Disease Improving Global Outcomes (KDIGO) guidelines, meeting one of the following: an increase in serum creatinine > 26.5 μmol/L within 48 hours; or serum creatinine ≥ 1.5× baseline within 7 days; urine output < 0.5 mL/kg/h for at least 6 hours. (b) Post-AKI renal biopsies, stratified into 2 groups based on time from AKI onset: an “early” group (biopsy 2–15 days post-AKI) and a “late” group (biopsy > 15 days after AKI).

The healthy control group was composed of patients with kidney tumors from the urology department, with histologically normal kidney tissues sampled ≥ 5 cm from tumor margins. Inclusion criteria for the control group were as follows: (a) GFR greater than 90 mL/min/1.73 m^2^; (b) urinary albumin excretion rate (AER) less than 30 mg/d or 20 mg/L, or albumin-to-creatinine ratio (ACR) <30 mg/g; and (c) normal blood pressure, liver function, and complete blood count. Exclusion criteria included: (a) history of metabolic diseases; (b) recent history of nephrotoxic medications; and (c) history of cardiovascular or cerebrovascular diseases. Following screening, 7 post-AKI biopsies (2 early, 5 late) and 7 control biopsies were included.

### Animals and animal care.

All animal experiments were conducted at Anhui Medical University (Anhui, China) under controlled conditions: temperature 22.5°C ± 0.5°C, humidity 50% ± 5%, a 12-hour light/dark cycle, and ad libitum access to water and a standard murine diet.

To establish the I/R-induced AKI-to-CKD mouse model, bilateral renal pedicles were clamped for 30 minutes using nontraumatic microaneurysm clamps (Roboz) to induce ischemia, followed by reperfusion. Kidneys were repositioned, and incisions were closed with absorbable sutures and wound clips. Sham surgeries were performed on control mice (*n* = 6–8). GFR was determined by measuring the transcutaneous elimination of FITC-sinistrin as described previously (39). Briefly, under brief isoflurane anesthesia, a fluorescence detector (MediBeacon) was placed on a depilated dorsal region, and FITC-sinistrin (50 mg/kg) was injected i.v. via the tail vein. Following anesthesia with sodium pentobarbital (50 mg/kg, i.p.), kidney and blood samples were collected for further experiments.

Eight-week-old male C57BL/6J mice were randomly divided into 2 groups: (a) mice injected with the HBAAV2/9-Null (EV, *n* = 6–8) and (b) mice injected with the HBAAV2/9-mmu_circ_0000465 (*CircNsd1*-OE, *n* = 6–8). A recombinant adeno-associated virus containing the mouse *CircNsd1*-OE gene was purchased from Hanheng Company (Hanheng Biotechnology Co.). The 150 UL of 1.7 × 10^12^ vg/mL adeno-associated virus 2/9 carrying the *CircNsd1*-OE or EV was injected by tail vein in mice (Supplemental Table 2). Two weeks after AAV2/9 administration, renal ischemia was induced by clamping the renal pedicle for 30 minutes. After removing the clamps to allow reperfusion, the kidneys were repositioned into the peritoneal cavity. At 12 weeks of age, mice were anesthetized with sodium pentobarbital (50 mg/kg, i.p.), and kidney tissues and blood samples were collected for analysis.

### Reagents and materials.

The following antibodies were used: anti–BACTIN antibody (Proteintech, 66009-1-Ig), anti-ACSL4 antibody (Santa Cruz Biotechnology, A0223), anti-GPX4 antibody (HUABIO, ET1706-45), anti-CD28K antibody (Proteintech, 66394-1-Ig), anti-AQP3 antibody (Santa Cruz Biotechnology, sc-518001), anti-SLC39A14 antibody (Proteintech, 26540-1-AP), anti–mouse Flag (Proteintech, 10024351), anti-H3K36me1 (Abcam, ab9048), anti-H3K36me2 (Abcam, ab9049), anti-H3K36me3 (Abcam, ab9050), anti–fluorescein labeled lotus tetragonolobus lectin (LTL) antibody (Vector Laboratories, FL-1321), anti-F4/80 antibody (Cell Signaling Technology, D2S9R), anti-IgG antibody (Cell Signaling Technology, 38191s-9), anti-ASMA (ZSbio, Kit-0006), and anti-CD68 antibody (Abcam, ab955). Masson’s trichrome staining kits were procured from Zhuhai Besso Biotechnology Institute. The PAS kits were obtained from Solarbio.

### Cell culture.

Human kidney tubular epithelial cell lines (HK-2 cells) were provided by Huiyao Lan (the Chinese University of Hong Kong, Hong Kong, China). The Cells were cultured in DMEM/F12 medium supplemented with 5% FBS (Gibco) at 37°C in a humidified atmosphere with 5% CO_2_. Following serum starvation, cells were transferred to an anoxic chamber (94% N_2_, 5% CO_2_, 1% O_2_) for 24 hours, before being returned to normoxic conditions. At 48 hours after reoxygenation, cells were harvested and analyzed for ferroptosis markers, oxidative stress, and inflammatory responses using real-time PCR and related assays.

### RNA extraction and real-time PCR.

Total RNA was extracted from kidney homogenates and HK-2 cells using TRIzol reagent (Takara) following the manufacturer’s instructions. RNA concentrations were determined using a NanoDrop 2000 Spectrophotometer (Thermo Fisher Scientific). cDNA was synthesized from total RNA using RealMasterMix (Yeasen) with nuclease-free water. Real-time PCR was performed using the Hieff UNICON qPCR SYBR Mix (Yeasen), as previously described (40, 41). Amplification and detection of cDNA were carried out using a Thermal Cycler system (CFX-6, Bio-Rad). β-Actin served as an internal reference gene for normalization, and the data are expressed as the mean ± SEM. Primer sequences used in this study are detailed in Supplemental Table 1. The relative quantification of gene expression was calculated using the 2^–ΔΔCt^ method.

### Dual-luciferase reporter assay.

Predicted sequences for the IRES were cloned into the Luc-IRES-Report vector (Hanbio). The sequence used was: 5′-TAAGAAAAAGTCTACGCCACTGAAGTATGAAGTTGGAGATCTCATCTGGGCAAAATTCAAGAGACGCCCATGGTGGCCCTGCAGGATTTGTTCTGATCCGTTGATTAACACACATTCAAAAATG-3′. Luciferase activity was measured using the Dual-Glo Luciferase Assay Kit (Promega) to quantify firefly and Renilla luciferase activities, following the manufacturer’s protocol (42, 43).

### Immunoprecipitation.

HK-2 Cells were harvested and washed thrice with ice-cold PBS as previously described (44). The cells were lysed in IP buffer (Beyotime Biotechnology) supplemented with a complete protease inhibitor cocktail (PIC) (Yeasen) on ice for 30 minutes. Lysates were collected, and immunoprecipitation was performed using anti-Flag Nanoab Magarose beads (NuoyiBio) by incubating with protein samples at 4°C for 12 hours. The resultant immunoprecipitated proteins were analyzed using gel electrophoresis and LC-MS/MS for identification and quantification, as reported previously (45).

### LC-MS/MS.

Immunoprecipitated proteins extracted from SDS-PAGE gels were subjected to enzymatic digestion, following established protocols. Gel pieces were washed with 50% acetonitrile (ACN) for 30 minutes, followed by a second wash with 100% ACN for an additional 30 minutes. After reduction and alkylation, the proteins were digested with trypsin at 37°C for 12 hours. The supernatant containing the digested peptides was collected and processed for desalination using an HPLC buffer system and analyzed by LC-MS/MS on Easy-nLC1200 chromatograph (Thermo Fisher Scientific) coupled to a Q Exactive plus mass spectrometer (Thermo Fisher Scientific). Data analysis was performed using Proteome Discoverer 2.4, incorporating the predicted sequence of NSD1-916aa and searching against the UniProt-reviewed Homo sapiens database. The experiments were conducted by OE Biotechnology Company, with data deposited in the National Genomics Data Center.

### Immunofluorescence.

Cells were fixed with 4% PFA for 5 minutes, rinsed with PBS, and blocked with 10% donkey serum for 60 minutes at 37°C. Subsequently, the cells were incubated with primary antibodies against α-SMA/Acsl4/SLC39A14 overnight at 4°C. After primary antibody incubation, the cells were treated with a FITC-conjugated secondary antibody (Bioss). Nuclei were counterstained with DAPI. After a final PBS wash, the sections were scanned using an automatic digital slide scanner (Pannoramic Scan).

### Lentivirus package and transfection of CircNSD1 overexpression.

The *circNSD*1-*FLAG* or control sequence was cloned into the pHBLV-CMV-circ-EF1-puro vector (Supplemental Table 3). This recombinant vector, along with packaging plasmids psPAX2 and pMD2.G, was cotransfected into HEK293T cells at a ratio of 4:3:1. After 48 hours, lentivirus particles were harvested from the culture supernatant and filtered. HK-2 cells or 293T cells were infected with the lentivirus, and stable cell lines were established by selection with puromycin (10 μg/mL) at 48 hours after infection.

### ChIP-qPCR.

To confirm that H3k36me1, H3k36me2, and H3k36me3 protein directly binds to the promoter of *ACSL4* and *SlC39A14*, ChIP assays were performed using the Enzymatic ChIP kit (Cell Signaling Technology). Cells were initially fixed with a crosslinking agent to stabilize protein-DNA interactions. Following crosslinking, cells were washed twice with cold PBS to remove excess crosslinking agent; they were then rinsed once with 2 mL of cold PBS supplemented with 10 μL of PIC to prevent proteolysis. Cells were subsequently lysed, and nuclei were isolated by centrifugation at 16,000*g*. The crosslinked chromatin was then fragmented by ultrasonication into DNA fragments ranging from 150 to 900 bp. ChIP was conducted by incubating samples with antibodies specific to anti-p65 or a nonspecific IgG (as a negative control) overnight at 4°C. DNA samples were purified using magnetic beads and subjected to ChIP–quantitative PCR (ChIP-qPCR) using the following primers: *ACSL4*, forward (F), 5′-GCGGGGATCTTAACGCAGTA-3′ and reverse (R), 5′-GTTCCCCTCGGGTCATCTTC-3′; *SlC39A14*, F, 5′-CCTCGGAACTTCTTCACCCC-3′ and R, 5′-TCCCACAATGATCAGACGCC-3′; *LPCAT3*, F, 5′-AGAGGAAATGCTAGGCCCGT-3′ and R, 5′-GTGTGCGCGAATCTGTGTTC-3′; and *SAT1*, F, 5′-CCAGTAGGGTTTCCGCCAAG-3′ and R, 5′-TGAACCCGGAGGACAAAAGT-3′.

### Untargeted LC/MS lipidomics analysis.

Lipids were extracted from H/R-treated control and circNSD1-overexpressing cells using a standardized protocol. Quality control (QC) volume was prepared by pooling equal volumes of extracts from all samples, ensuring that the volume of each QC sample was equivalent to that of the individual samples. Subsequently, the samples were subjected to separation using a Nexera UPLC system (Shimadzu) and analyzed via a Q Exactive mass spectrometer (Thermo Fisher Scientific). The LC/MS detection was performed at Lumingbio Company. A supervised multiple regression OPLS-DA model was applied to identify differential lipid metabolites. Permutation testing with 200 iterations was conducted to mitigate overfitting risks. All experiments were conducted by OE Biotechnology Company and data are available at the National Genomics Data Center (https://ngdc.cncb.ac.cn/omix/release/OMIX007361).

### Kidney histology.

Kidney tissue sections were prepared from paraffin-embedded samples and subjected to IHC staining. Antigen retrieval was performed using a microwave-based method. Sections were incubated with rabbit-derived primary antibodies against ASMA, F4/80, or CD68 for 24 hours at 4°C, followed by incubation with appropriate secondary antibodies for 30 minutes at 37°C. Staining was visualized using 3,3’-diaminobenzidine (DAB) as the chromogen. Slides were scanned using a Pannoramic Scan digital slide scanner (3D Histech).

### Evaluation of renal damage.

Human and mouse kidney tissues were fixed in 4% paraformaldehyde (PFA), dehydrated, and embedded in paraffin. Tissue sections (5 μm thick) were stained with H&E to assess morphology, PAS to evaluate tissue damage, and Masson’s trichrome to quantify fibrosis, following the manufacturer’s protocol (46).

Renal damage was assessed using PAS- and H&E-stained kidney specimens. Parameters evaluated included tubular detachment, tubular dilation, protein casts, and brush border damage. Six random fields per section were analyzed, with results expressed as the percentage of affected area per whole section. The extent of tubular detachment, proximal tubular dilation, and brush border damage was scored on a scale from 0 to 10, with the following categorization: 0–1 indicating none (0%), 1–2 indicating less than 11%, 2–4 indicating 11%–25%, 4–6 indicating 26%–45%, 6–8 indicating 46%–75%, and 8–10 indicating greater than 75% (47).

### Detection of circNSD1 levels in serum.

To measure circNSD1 levels in serum, samples from patients and controls were processed as follows. Serum was centrifuged at 2,000*g* for 30 minutes at 4°C, and the supernatant was collected. The supernatant was further centrifuged at 12,000*g* at 4°C for 45 minutes, filtered through a 0.22 μm filter (MilliporeSigma), and ultracentrifuged at 110,000*g* for 70 minutes at 4°C (Beckman Ultracentrifuge). The pellet was washed with PBS, recentrifuged at 110,000*g* for 70 minutes, and resuspended in 500 μL of TRIzol reagent. Total RNA was extracted, and circNSD1 levels were quantified by real-time PCR according to standard protocols.

### Statistics.

Data are presented as the mean ± SEM (38). Statistical significance was determined using 1-way ANOVA, followed by Tukey’s post hoc test for multiple comparisons. Additionally, 2-tailed Student’s *t* test were also performed to compare the means of 2 groups when necessary, as performed with GraphPad Prism version 5.0 software (GraphPad Software).

### Study approval.

The clinical studies were performed in accordance with the principles of the Declaration of Helsinki after obtaining informed consent from the patients. It was approved by the Research Ethics Committee of the First Affiliated Hospital of Anhui Medical University (no. 2023187). All animal experiments were approved by the Anhui Medical University Ethical Committee on Animal Experiments (no. LLSC20211279).

### Data availability.

The datasets used or analyzed in the study are available from the Supporting Data Values file. The high-throughput circRNA-Seq data in this publication have been deposited in the National Center for Biotechnology Information Gene Expression Omnibus (https://www.ncbi.nlm.nih.gov/geo/query/acc.cgi?acc=GSE139107). The MS proteomics data have been deposited to the ProteomeXchange Consortium via the PRIDE partner repository with the dataset identifier OMIX007182, OMIX007357, and OMIX007361 (https://ngdc.cncb.ac.cn/omix/release/OMIX007182; https://ngdc.cncb.ac.cn/omix/release/OMIX007357; https://ngdc.cncb.ac.cn/omix/release/OMIX007361). All original data are accessible through the corresponding author upon reasonable request.

## Author contributions

LG, JZ, CC and SZ contributed methodology, investigation, review and editing, writing of the original draft, and formal analysis. XW, GT, SW, Y Wang, and XL contributed review and editing as well as methodology. LJ and Y Wu contributed conceptualization, supervision, project administration, and funding acquisition. All authors reviewed the manuscript.

## Supplementary Material

Supplemental data

Unedited blot and gel images

Supporting data values

## Version 1 07/15/2025

In-Press Preview

## Version 2 08/22/2025

Electronic publication
