# Supplementary material for: NSD1-916aa encoded by CircNSD1 contributes to AKI-to-CKD transition through inducing ferroptosis in tubular epithelial cells
Source: JCI Insight. 2025 Jul 15;10(16):e189130. doi: 10.1172/jci.insight.189130 (PMC12406731; doi:10.1172/jci.insight.189130)
Supplement: Supplemental data [file jciinsight-10-189130-s257.pdf]

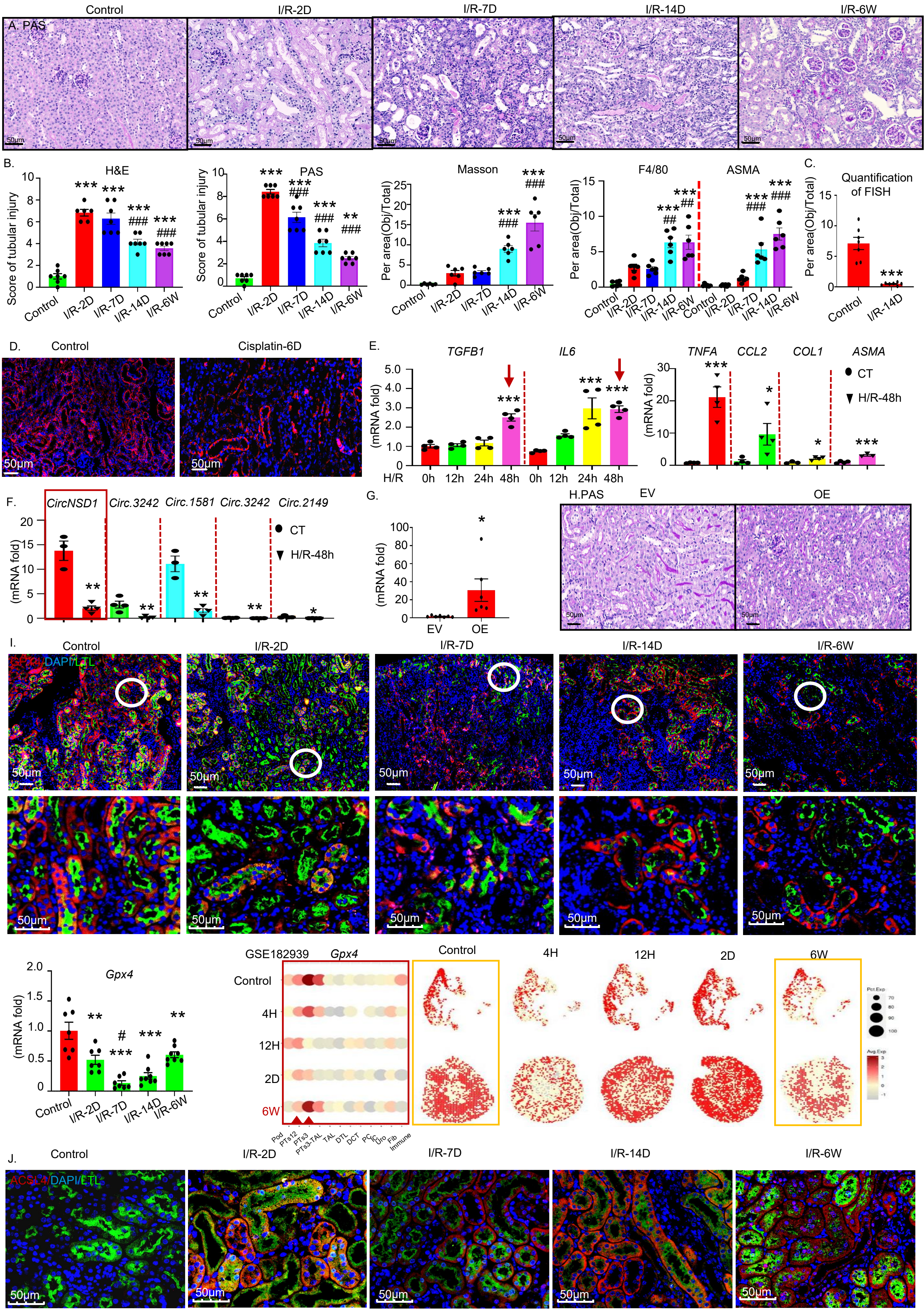

Supplemental Figure1

A. *CircNsd1*-FLAG

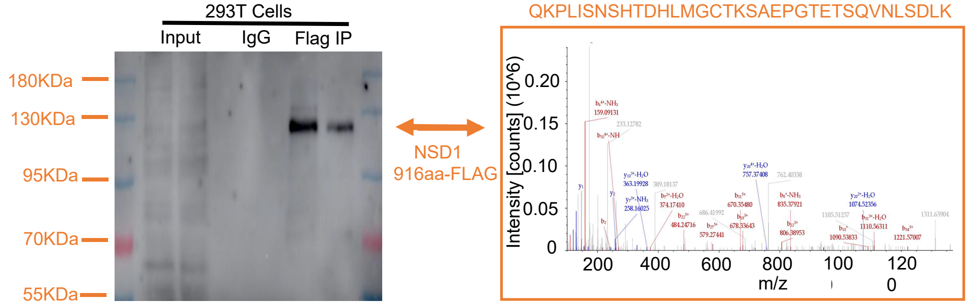

B. Results of LC/MS in HK-2 cells

Gene Symbol	Coverage [%]	Peptides	MW [kDa]	AAs	Score Sequest HT
CAND1	54	60	136.3	1230	332.04
CCDC47	51	27	55.8	483	326.15
SRRT	48	52	100.6	876	306.31
FLNA	33	63	280.6	2647	228.42
RBM10	42	45	103.5	930	253.82

C. Results of LC/MS in 293T cells

Gene Symbol	Coverage [%]	Peptides	MW [kDa]	AAs	Score Sequest HT
CAND1	35	39	136.3	1230	139.25
TJP2	33	35	133.9	1190	113
SUPT5H	34	27	120.9	1087	104.89
NCKAP1	32	34	128.7	1128	79.59
CTNND1	39	29	108.1	968	83.82

Supplemental Figure2

Supplement Table 1: Primer sequences used in real-time PCR

	Forward (5’-3’)	Reverse (5’-3’)
Mice-tgfb1	GCAACAATTCCTGGCGTTAC	GCTGAATCGAAAGCCCTGTA
Mice-col1	GGTCTTGGTGGTTTTGTATTCG	AACAGTCGCTTCACCTACAGC
Mice-αsma	TCCTGTTTCGGGAGCAGAAC	AGCTGGCCGTTCACTCTAAC
Mice-tnfα	CATCTTCTCAAAATTCGAGTGACAA	TGGGAGTAGACAAGGTACAACCC
Mice-ccl2	GTCTGTGCTGACCCCAAGAAG	TGGTTCCGATCCAGGTTTTTA
Mice-il6	TCCAGTTGCCTTCTTGGGAC	GTGTAATTAAGCCTCCGACTTG
Mice-β-actin	GATATCGCTGCGCTGGTCG	CCACGATGGAGGGGAATACAG
Mice-Circnsd1	TGACCTCCATAGAAGACACCGGGA TCCAAGTGCTGAGATTACAGGCGT	ATCGATAAGCTTGATATCGAATTCTG CTGGGATTACAGGTGTGAGCTAC
Human-RF1	TGGTCGTGGGGACTGTTCTA	CCCCCACTAGGGCTAGGATT
Human-RF2 (CircNSD1)	CTGAGCTCCCTGAACCAGTGTC	CACCATGGGCGTCTCTTGAA
Human-TGFB1	AGCTGTACATTGACTTCCGCA	AGAAGTTGGCATGGTAGCCC
Human-COL-1	TCTAGACATGTTCAGCTTTGTGGAC	TCTGTACGCAGGTGATTGGTG
Human-ASMA	GACAATGGCTCTGGGCTCTGTAA	CTGTGCTTCGTCACCCACGTA
Human-TNFA	CTCACATACTGACCCACGGC	CCGGATCATGCTTTCAGTGC
Human-CCL2	AGAGGCTGAGACTAACCCAGA	TTTCATGCTGGAGGCGAGAG
Human-IL-6	GGTACATCCTCGACGGCATCT	GTGCCTCTTTGCTGCTTTCAC
Human-β-ACTIN	CGCCGCCAGCTCACCATG	CACGATGGAGGGGAAGACGG

Supplement Table 2: Overexpression of Mice CircNSD1

Circ Nsd1 Overexpression		Sequences (5'-3')
OE	PAAV-RC CircNSD1	TGTCAACCGAAGAAAAAGTCTACACCACTGAAATATGAAGTTGGA<br>GATCTTATTTGGGCAAAATTCAAGAGACGCCCTTGGTGGCCCTGCA<br>GGATCTGTTCTGATCCATTGATTAATACACACTCAAAAATGAAAGT<br>TGCCAATAGGAGGCCATATCGCGAATACTACGTAGAGGCTTTTGG<br>AGACCCTTCTGAAAAAGCCTGGGTGGCTGGAAAAGCAATCGTCAT<br>GTTTGAAGGCAGACATCAATTTGAAGAACTACCTGTCCTTAGGAA<br>AAGAGGGAAACAGAAAGAAAAAGGATATAGGCATAAGGTTCCTC<br>AGAAGATTTTGAGTAAATGGGAAGCCAGCGTTGGTCTTGCCGAGC<br>AATATGATGTTCCCAAAGGCTCTAAGAACCAAAAGTGTGTCAGTA<br>GCTCAGTCAAGTTGGACAGTGAGGAAGATATGCCATTTGAGGACT<br>GTACAAACGATCCTGATTCAGAACACCTGTTGCTTAATGGCTGCTT<br>GAAGTCTCTGGCTTTTGATTCTGAGCATTCTGCAGATGAAAAGGAA<br>AAGCCCTGTGCCAAGTCTCGAGTTAGAAAAAGCTCTGATAATATA<br>AAAAGGACTAGTGTGAAAAAGGATCTGGTGCCATTTGAATCACGT<br>AAAGAAGAACGGAGGGGGAAAATTCCAGACAACCTTGGCCTAGA<br>CTTTATCTCTGGGGGTGTATCTGATAAACAGGCTTCTAATGAACTT<br>TCCAGGATAGCAAACAGCCTCACAGGGTCTAGCACTGCACCAGGA<br>AGTTTCCTCTTTTCTTCCAGTGTACAGAACACTGCTAAGACAGACT<br>TTGAGACTCCAGATTGCGATTCCTTATCAGGTTTGTCTGAGAGTGC<br>TTTAATCTCTAAACATTCTGGAGAGAAAAAGAAACTCCAACCAGG<br>CCAGGTCTGCAGCTCAAAAGTACAGCTCTGCTACGTTGGAGCTGG<br>TGATGAGGAAAAGCGAAGCAATTCTGTCAGTGTGAGTACCACTTC<br>TGATGATGGCTGCAGTGACCTGGATCCTACAGAGCACAACTCAGG<br>ATTCCAGAACAGTGTCCTTGGAATTACAGATGCTTTTGATAAAACA<br>GAGAATGCTTTATCGGTGCACAAAAATGAAACACAGTATTCTAGA<br>TATCCTGTCACAAACAGGATAAAAGAGAAACAGAAATCCCTCATT<br>ACTAATTCCCATGCAGACCATTTAATGGGTTCTACCAAGACAATGG<br>AGCCTGAAACTGCTGAGTTGTCTCAGGTCAATCTTTCTGATCTTAA<br>AATTAGTAGTCCTATTCCCAAACCCCAACCAGAATTTAGGAATGAT<br>GGTCTCACTACAAAATTCAGCGCGCCACCAGGCATTCGTAATGAA<br>AATCCACTGACAAAGGGTGGGCTTGCAAATCAAACTCTATTACCT<br>CTGAAATGCAGACAGCCCAAGTTTCGTAGTATAAAATGCAAGCAT<br>AAAGAAAGCCCAGCTGTTGCAGAAACCTCAGCTACAAGTGAGGAC<br>CTCAGTTTGAAATGCTGTTCTTCTGATACCAATGGCTCTCCTTTGGC<br>CAATATATCCAAAAGTGGGAAAGGAGAGGGGCTAAAACTACTGA<br>ACAATATGCATGAGAAAACCAGAGATTCTAGTGACATAGAAACAG<br>CAGTGGTGAAGCACGTTCTTTCGGAACTGAAGGAGCTCTCGTACA<br>GATCATTAAGTGAAGACGTAAGTGATTCTGGAACGGCAAAGGCAT

		CAAAGCCATTACTGTTTTCTTCTGCTTCTAGTCAGAATCATATACC<br>AATTGAACCAGATTACAAGTTTAGCACATTGCTGATGATGCTGAA<br>GGATATGCATGACAGTAAGACAAAGGAGCAGCGGTTAATGACTGC<br>TCAAAATCTGGCTTCCTACAGGACTCCAGATCGTGGGGACTGTTCT<br>TCTGGTAGCCCTGTAGGGACATCAAAAGTTTTGGTGTTAGGAAGCT<br>CCACACCCAATTCTGAAAAGCCTGGAGATAGCACTCAGGACTCAG<br>TTCACCAGAGTCCTGGTGGGGGTGACTCTGCCTTGTCTGGAGAGTT<br>GTCTTCTTCCCTGTCCAGCTTAGCTTCTGACAAAAGAGAGCTCCCT<br>GCTTGTGGTAAAATTCGCTCTAACTGTATCCCAAGGCGCAACTGTG<br>GCCGAGCAAAGCCATCATCCAAGTTGAGAGAAACCATTTCAGCCC<br>AGATGGTAAAACCTTCAGTAAACCCAAAAGCCTTAAAGACTGAGC<br>GGAAGAGAAAGTTCAGCCGACTTCCAGCTGTGACACTTGCTGCTA<br>ACAGACTGGGAAACAAAGAAAGTGGATCAGTGAATGGCCCATCC<br>AGGGGTGGGGCTGAAGATCCTGGTAAAGAAGAGCCTCTGCAACAA<br>ATGGACCTTTTAAGAAATGAAGACACACATTTTTCAGATGTACATT<br>TTGATAGCAAGGCTAAACAATCGGACCCTGATAAAAATCTTGAAA<br>AAGAACCTTCTTTTGAGAACAGAAAAGGCCCAGAGTTGGGCTCTG<br>AAATGAACACTGAGAATGATGAACTCCATGGTGTTAATCAGGTGG<br>TGCCTAAAAAGCGGTGGCAGCGATTAAACCAAAGGCGCCCTAAGC<br>CCGGAAAGCGTGCTAACAGATTTAGGGAGAAAGAAAACTCGGAG<br>GGTGCTTTTGGAGTCTTACTTCCTGCTGACGCTGTGCAGAAGGCCC<br>GGGAAGACTACTTAGAACAAAGAGCTCCTCCTACAAGTAAACCTG<br>AGGACTCAGCAGCAGATCCCAATCATGGCAGCCACTCTGAATCGG<br>TTGCGCCTCGGCTGAATGTTTGTGAGAAGTCCAGTGTTGGCATGGG<br>TGATGTAGAAAAGGAGACAGGAATTCCCAGTTTGATGCCACAGAC<br>CAAGCTCCCTGAGCCAG

Supplement Table 3: Overexpression of Human CircNSD1-Flag

Circ Nsd1 Overexpression		Sequences (5’-3’)
OE	PLCDH-CircNSD1-Flag	TGTCAACCgactacaaggatgacgatgacaaggattacaaagacgacgatgataaggactataaggat<br>gatgacgacaaaTAAGAAAAAGTCTACGCCACTGAAGTATGAAGTTGGA<br>GATCTCATCTGGGCAAAATTCAAGAGACGCCCATGGTGGCCCTGC<br>AGGATTTGTTCTGATCCGTTGATTAACACACATTCAAAAATGAAAG<br>TTTCCAACCGGAGGCCCTATCGGCAGTACTACGTGGAGGCTTTTGG<br>AGATCCTTCTGAGAGAGCCTGGGTGGCTGGAAAAGCAATCGTCAT<br>GTTTGAAGGCAGACATCAATTCGAAGAGCTACCTGTCCTTAGGAG<br>AAGAGGGAAACAGAAAGAAAAAGGATATAGGCATAAGGTTCCTC<br>AGAAAATTTTGAGTAAATGGGAAGCCAGTGTTGGACTTGCAGAAC<br>AGTATGATGTTCCCAAGGGGTCAAAGAACCGAAAATGTATTCCTG<br>GTTCAATCAAGTTGGACAGTGAAGAAGATATGCCATTTGAAGACT<br>GCACAAATGATCCTGAGTCAGAACATGACCTGTTGCTTAATGGCT<br>GTTTGAAATCACTGGCTTTTGATTCTGAACATTCTGCAGATGAGAA<br>GGAAAAGCCTTGCGCTAAATCTCGAGCCAGAAAGAGCTCTGATAA<br>TCCAAAAAGGACTAGTGTGAAAAAGGGCCACATACAATTTGAAGC<br>ACATAAAGATGAACGGAGGGGAAAGATTCCAGAGAACCTTGGCCT<br>AAACTTTATCTCTGGGGATATATCTGATACGCAGGCCTCTAATGAA<br>CTTTCCAGGATAGCAAATAGCCTCACAGGGTCCAACACTGCCCCA<br>GGAAGTTTTCTGTTTTCTTCCTGTGGAAAAAACACTGCAAAGAAAG<br>AATTTGAGACTTCAAATGGTGACTCTTTATTGGGCTTGCCTGAGGG<br>TGCTTTGATCTCAAAGTGTTCTCGAGAGAAGAATAAACCCCAACG<br>AAGCCTGGTGTGTGGTTCAAAAGTGAAGCTCTGCTATATTGGAGC<br>AGGTGATGAGGAAAAGCGAAGTGATTCCATTAGTATCTGTACCAC<br>TTCTGATGATGGAAGCAGTGACCTGGATCCCATAGAACACAGCTC<br>AGAGTCTGATAACAGTGTCCTTGAAATTCCAGATGCTTTCGATAGA<br>ACAGAGAACATGTTATCTATGCAGAAAAATGAAAAGATAAAGTAT<br>TCTAGGTTTGCTGCCACAAACACTAGGGTAAAAGCAAAACAGAAG<br>CCTCTCATTAGTAACTCACATACAGACCACTTAATGGGTTGTACTA<br>AGAGTGCAGAGCCTGGAACCGAGACGTCTCAGGTTAATCTCTCTG<br>ATCTGAAGGCATCTACTCTTGTTCACAAACCCCAGTCAGATTTTAC<br>AAATGATGCTCTCTCTCCAAAATTCAACCTGTCATCAAGCATATCC<br>AGTGAGAACTCGTTAATAAAGGGTGGGGCAGCAAATCAAGCTCTA<br>TTACATTCGAAAAGCAAACAGCCCAAGTTCCGAAGTATAAAGTGC<br>AAACACAAAGAAAATCCAGTTATGGCAGAACCCCCAGTTATAAAT<br>GAGGAGTGCAGTTTGAAATGCTGCTCTTCTGATACCAAAGGCTCTC<br>CTTTGGCCAGCATTTCTAAAAGTGGAAAGTGGATGGTCTAAAAC<br>TACTGAACAATATGCATGAGAAAACCAGGGATTCAAGTGACATAG<br>AAACAGCAGTGGTGAAACATGTTTTATCCGAGTTGAAGGAACTCT

		CTTACAGATCCTTAGGTGAGGATGTCAGTGACTCTGGAACATCAA AGCCATCAAAACCATTACTTTTCTCTTCTGCTTCTAGTCAGAATCA CATACCTATTGAACCAGACTACAAATTCAGTACATTGCTAATGATG TTGAAAGATATGCATGATAGTAAGACGAAGGAGCAGCGGTTGATG ACTGCTCAAAACCTGGTCTCTTACCGGAGTCCTGGTCGTGGGGACT GTTCTACTAATAGTCCTGTAGGAGTCTCTAAGGTTTTGGTTTCAGG AGGCTCCACACACAATTCAGAGAAAAAGGGAGATGGCACTCAGA ACTCCGCCAATCCTAGCCCTAGTGGGGGTGACTCTGCATTATCTGG CGAGTTGTCTGCTTCCCTACCTGGCTTACTGTCCGACAAGAGAGAC CTCCCTGCTTCTGGTAAAAGTCGTTCAGACTGTGTTACTAGGCGCA ACTGTGGACGATCAAAGCCTTCATCCAAATTGCGAGATGCTTTTTC AGCCCAAATGGTAAAGAACACAGTGAACCGTAAAGCCTTAAAGAC CGAGCGCAAAAGAAAACTGAATCAGCTTCCAAGTGTGACTCTTGA TGCTGTACTGCAGGGAGACCGAGAACGTGGAGGTTCATTGAGAGG TGGGGCAGAAGATCCTAGTAAAGAGGATCCCCTTCAGATAATGGG CCACTTAACAAGTGAAGATGGTGACCATTTTTCTGATGTGCATTTC GATAGCAAGGTTAAGCAATCTGATCCTGGTAAAATTTCTGAAAAA GGACTCTCTTTTGAAAACGGAAAAGGCCCAGAGCTGGACTCTGTA ATGAACAGTGAGAATGATGAACTCAATGGTGTAAATCAAGTGGTG CCTAAAAAGCGGTGGCAGCGTTTAAACCAAAGGCGCACTAAACCT CGTAAGCGCATGAACAGATTTAAAGAGAAAGAAAACTCTGAGTGT GCCTTTAGGGTCTTACTTCCTAGTGACCCTGTGCAGGAGGGGCGGG ATGAGTTTCCAGAGCATAGAACTCCTTCAGCAAGCATACTTGAGG AACCACTGACAGAGCAAAATCATGCTGACTGCTTAGATTCAGCTG GGCCACGGTTAAATGTTTGTGATAAATCCAGTGCCAGCATTGGTG ACATGGAAAAGGAGCCAGGAATTCCCAGTTTGACACCACAGGCTG AGCTCCCTGAACCAG

Figure S1.

A. PAS Staining in kidney tissues from control and I/R mice. B. Quantitative analysis of pathological images in Fig 1E-H. Data are shown as mean±SEM, **p < 0.01, ***p < 0.001 compared with the control group by one-way ANOVA with Tukey's post hoc test. ##p < 0.01, ###p < 0.001 compared with the I/R-2D group by one-way ANOVA with Tukey's post hoc test. C. Quantification of FISH in Fig 2C. Data are shown as mean±SEM, ***p < 0.001 compared with the Control group by Student's t-test. D. FISH assay for *CircNSD1*. Scale bar: white 50 μm. E. Real-time PCR analysis of *TGFB1* and *IL6* expression in HK-2 cells during different periods of H/R induction.

Data are shown as mean±SEM, ***p < 0.001 compared with the 0h group by one-way ANOVA with Tukey's post hoc test. Real-time PCR analysis of inflammation-related genes (*TNFA*, *CCL2*) and fibrosis-related genes (*COL1* and *ASMA*) in HK-2 cells after 48h H/R induction. Data are shown as mean±SEM, *p < 0.05, ***p < 0.001 compared with the CT group by Student's t-test. F. Real-time PCR analysis showing the expression of different circRNAs in HK-2 cells after 48h H/R induction. Data are shown as mean±SEM, *p < 0.05, **p < 0.01 compared with the CT group by Student's t-test. G. Real-time PCR of *CircNsd1* in kidney tissues from control and CircNSD1-overexpressing mice. Data are shown as mean±SEM, *p < 0.05 compared with the EV group by Student's t-test. H. PAS staining was performed in kidney tissues from control and *CircNsd1*-overexpressing mice. I. Immunofluorescence staining of GPX4 and LTL in kidney tissues from 2 days to 6 weeks post- I/R injury. Scale bar: white 50μm; Relative expression of GPX4 detected by real time-PCR from 2 days to 6 weeks post- I/R injury. Data are shown as mean±SEM, **p < 0.01, ***p < 0.001 compared with the control group by one-way ANOVA with Tukey's post hoc test. #p < 0.05 compared with the I/R-2D group by one-way ANOVA with Tukey's post hoc test; Results of GPX4 expression levels in mouse kidneys during the transition from AKI to CKD, analyzed from the spatial transcriptomics dataset (GSE182939) on the KIT website. J. Immunofluorescence staining of ACSL4 and LTL in kidney tissues from 2 days to 6 weeks post- I/R injury. Scale bar: white 20 μm.

Figure S2.

A. Western blot analysis shows that NSD1-916aa in 293T cells and the MS results of 293T cells revealing unique sequences of NSD1-916aa. B. Results of LC/MS in HK-2 cells. C. Results of LC/MS in 293T cells.
