# Supplementary figures and images for: NSD1-916aa encoded by CircNSD1 contributes to AKI-to-CKD transition through inducing ferroptosis in tubular epithelial cells
Source: JCI Insight. 2025 Jul 15;10(16):e189130. doi: 10.1172/jci.insight.189130 (PMC12406731; doi:10.1172/jci.insight.189130)

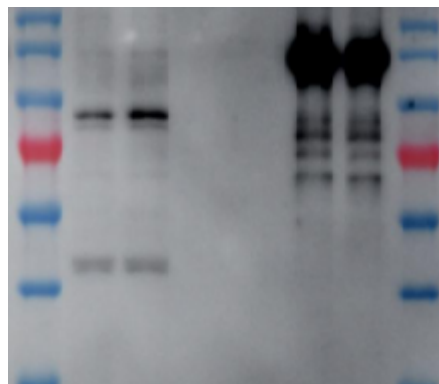

Supplement: Unedited blot and gel images [file jciinsight-10-189130-s258.pdf]
